# Inhibition of HIF-1α by Atorvastatin During ^131^I-RTX Therapy in Burkitt’s Lymphoma Model

**DOI:** 10.3390/cancers12051203

**Published:** 2020-05-11

**Authors:** Eun-Ho Kim, Hae Young Ko, A Ram Yu, Hyeongi Kim, Javeria Zaheer, Hyun Ji Kang, Young-Cheol Lim, Kyung Deuk Cho, Hyun-Yoo Joo, Min Kyoung Kang, Jae Jun Lee, Seung-Sook Lee, Hye Jin Kang, Sang Moo Lim, Jin Su Kim

**Affiliations:** 1 Division of Radiation Biomedical Research, Korea Institute of Radiological and Medical Sciences, 75 Nowon-ro, Nowon-gu, Seoul 01812, Korea; eunhokim8@gmail.com (E.-H.K.); hyunyoo@kirams.re.kr (H.-Y.J.); 2 Department of Biochemistry, School of Medicine, Catholic University of Daegu, 33, 17-gil, Duryugongwon-ro, Nam-gu, Daegu 705-718, Korea; 3 Division of RI Application, Korea Institute of Radiological and Medical Sciences (KIRAMS), 75 Nowon-ro, Nowon-gu, Seoul 01812, Korea; HYKO23@yuhs.ac (H.Y.K.); erwin.hyeongi@gmail.com (H.K.); javeria24@kirams.re.kr (J.Z.); hjk@ibs.re.kr (H.J.K.); zerofe0701@gmail.com (Y.-C.L.); bluesea299@daum.net (K.D.C.); smlim328@kirams.re.kr (S.M.L.); 4 Department of Nuclear Medicine, Yonsei University College of Medicine, Seoul 03722, Korea; 5 Laboratory Animal Center, Osong Medical Innovation Foundation, Osong, Chungbuk 28159, Korea; kykatr@kbiohealth.kr (A.R.Y.); kmkccm@kbiohealth.kr (M.K.K.); jlee@kbiohealth.kr (J.J.L.); 6 Radiologcial and Medico-Oncological Sciences, University of science and technology (UST), Seoul 01812, Korea; 7 Department of Pathology, Korea Cancer Center Hospital, Korea Institute of Radiological and Medical Sciences, Seoul 01812, Korea; sslee@kirams.re.kr; 8 Division of Hematology/Oncology, Department of Internal Medicine, Korea Cancer Center Hospital, Korea Institute of Radiological and Medical Sciences, Seoul 01812, Korea; hyejin@kirams.re.kr; 9 Department of Nuclear Medicine, Korea Cancer Center Hospital, Korea Institute of Radiological and Medical Sciences, Seoul 01812, Korea

**Keywords:** HIF-1α, radioimmunotherapy, rituximab, atorvastatin, lymphoma, ^131^I, RIT, VEGF

## Abstract

Backgrounds: Radioimmunotherapy (RIT) serves as a targeted therapy for non-Hodgkin lymphomas (NHL). Although HIF(Hypoxia-inducible factors)-1α is an important biomarker during radiation therapy, its role in NHL is unclear. Atorvastatin (ATV) is used as a combination drug for chemotherapy. Methods: We investigated whether ATV downregulated tumor radio-resistance and enhanced the anticancer effect of ^131^I-RTX (rituximab) in Raji xenograft mouse models. First, the increased uptake and enhanced therapeutic effect of ^131^I-RTX by ATV was confirmed using molecular imaging in Raji xenograft subcutaneous model and orthotropic model with SPECT and IVIS images. Second, we examined the profile of differentially expressed miRNAs using miRNA array. Results: We found that miR-346 inhibited HIF-1α/VEGF (Vascular endothelial growth factor) during ATV combination therapy with ^131^I-RTX. The underlying mechanism of ATV involved induction of anti-angiogenesis and radiosensitivity by downregulating HIF-1α in Raji cells. Conclusion: Our findings suggested that combination therapy with ATV and ^131^I-RTX is a promising strategy for enhancing the potency of ^131^I-RTX therapy in poorly responding patients and those with radio-resistance.

## 1. Introduction

Radioimmunotherapy (RIT) using radiolabeled monoclonal antibodies served as a targeted therapy for the treatment of relapsed and refractory non-Hodgkin lymphomas (NHL) [1]. CD20 is an extracellular surface protein expressed in most human B-cell-lineage malignancies; anti-CD20 antibodies are known for their potential ability to target NHL [2]. Rituximab (RTX) is a monoclonal antibody for the CD20 antigen [3,4]. RIT using ^131^I-RTX has already been reported for relapsed or refractory indolent patients with NHL [5,6,7]. In RIT of NHL, the targeting properties of anti-CD20 antibodies are explored via conjugation of the antibodies with radioactive isotopes [8]. Despite the success of RIT, certain patients treated with conventional RIT still relapse. The response rate and response duration have been reported to increase during repeated RIT of ^131^I-RTX, but they were < 70% [9]. Most studies investigated diffuse large B cell lymphoma (DLBCL) among different NHL types [10,11]; however, the prognosis for Burkitt’s lymphoma (BL) was poor in elderly patients and those with relapsed disease [12,13]. 

Although HIF-1α is an important biomarker during radiation therapy in solid tumors, its role in NHL is unclear. Notably, it has been reported that overall survival of HIF-1α-positive patients was superior to that of HIF-1α-negative patients with DLBCL [14]. To the best of our knowledge, the role of HIF-1α in patients with BL has not been reported to date. Recently, we identified highly expressed HIF-1α in patients with BL using RNAseq database (http://www.ebi.ac.uk/gxa). 

Atorvastatin (ATV) is used to lower cholesterol in the treatment of hypercholesterolemia [15,16]. We previously demonstrated that ATV enhanced the anticancer effect when used in combination therapy with trastuzumab [17]. In this study, we investigated whether ATV downregulated tumor radio-resistance and enhanced the anticancer effect of ^131^I-RTX in BL Raji xenograft mouse models. We found that miR-346 inhibited HIF-1α/VEGF during ATV combination therapy with ^131^I-RTX. Moreover, the role of miRNAs, underlying mechanism of promoting radiosensitivity, and anti-angiogenic properties of ATV during ^131^I-RTX therapy were revealed. 

Our findings suggested that ATV + ^131^I-RTX therapeutic regimen is a promising strategy for enhancing the potency of ^131^I-RTX therapy in poorly responding patients and those with radio-resistance. The limitation of using ^131^I-RTX alone and strengths of our proposed therapeutic strategy are presented as a schematic in Figure S1a. 

## 2. Methods

### 2.1. Cell Culture

Raji cells, a human Burkitt lymphoma cell line, were obtained from American Type Culture Collection (ATCC, Manassas, VA, USA) and maintained in RPMI supplemented with 10% fetal bovine serum (FBS) and antibiotics (Sigma, St. louis, MO, USA). The cells were maintained at 37 °C in a humidified 5% CO_2_ incubator. MINO, U2932, and Jeko-1 (mantle cell lymphoma) cells were also maintained under the same condition. 

### 2.2. Cell Cycle Analysis

After 1 day of irradiation, the cells were harvested for analysis. The cells were pelleted by centrifugation at. 1200 rpm for 3 min, fixed by the dropwise addition of 5 mL ice-cold 70% ethanol with vortexing, and then incubated at 4 °C for at least 1 h. The fixed cells were pelleted by centrifugation, rinsed with PBS, and pelleted again. The cells were then incubated with propidium iodide ReadyProbes^TM^ reagent (2 drops/1 × 10^6^ cells, Thermo Fisher Scientific, Eugene, OR, USA) containing 100 mg RNase (1 mg/mL in PBS; Qiagen) at 37 °C for 30 min. Finally, 10,000 cells of each sample were analyzed by fluorescence-activated cell sorter (BD Biosciences, San Jose, CA, USA) and CellQuest software (BD Biosciences, San Jose, CA, USA) to determine their cell cycle stage.

### 2.3. Lymphoma Xenograft Subcutaneous Model

Five-week-old female NOD/SCID mice were obtained from Animal Resource Center (WA, Australia). The mice were maintained in temperature-controlled clean racks with a 12-h light/dark cycle and allowed to acclimatize for 1 week before the start of the experiment. Approximately 1 × 10^7^ Raji cells were injected subcutaneously into the right flank. When the tumor size reached approximately 200 mm^3^, the mice were randomized into each group (*n* = 4–5/group). The tumor size was measured at the indicated times by using a digital caliper, and the tumor volume was calculated using the formula width^2^ × length × 0.4. To monitor potential toxicity, body weight was measured. The mice were euthanized when the tumor size exceeded the volume of 1,500 mm^3^ or the body weight loss was > 20% of the original weight.

### 2.4. Conjugation of Alexa Fluor 488 to Rituximab

A solution of Alexa Fluor 488 (Invitrogen, Carlsbad, CA, USA) in dimethyl sulfoxide with 1% acetic acid was prepared. This solution was immediately added to 500 μL (10 mg/mL) dissolved in 1 M of sodium bicarbonate solution, pH 8.4. The solution was mixed thoroughly and left to stand for 1 h at room temperature. This reaction solution was purified by using a size exclusion PD-10 column (GE Healthcare, IL, USA) with phosphate-buffered saline (PBS) as the elution buffer. The protein concentration of the purified solution was quantified by using a Nano-drop spectrophotometer.

### 2.5. In Vivo Antibody Penetration Studies

When the tumor size reached ~200 mm^3^, Alexa488-RTX was intravenously injected as a single dose (150 μg), and ATV (12 μg/day in PBS) (approximately equivalent to 40 mg/day in human treatment) was administered via oral gavage for 5 days. After 5 days, the mice were exsanguinated by cardiac puncture and dissected. The tumors were isolated from the mice and immediately fixed with 4% paraformaldehyde overnight at 4 °C. Then, 8-μm tumor sections from three different regions were cut using a Leica CM 1850 cryostat (Leica microsystems, Wetzlar, Germany) to obtain representative sections throughout the tumor. After three washes with 200 μL PBS, TUNEL-positive cells were stained with Click-iT^®^ TUNEL Alexa Fluor^®^ 647 Imaging Assay kit (Invitrogen, Carlsbad, CA, USA). For antibody penetration, we imaged and calculated Alexa488-RTX in whole tumor images. And apoptotic cells was calculated by TUNEL assay. 

### 2.6. ^131^I-Radiolabeling with Rituximab

Pierce pre-coated iodination tubes (Thermo Scientific, Eugene, OR, USA) were used for ^131^I radiolabeling of RTX. ^131^I (100 μL; 59.2 MBq) was added in a pre-coated iodination tube and incubated for 10 min with shaking at 18–21 °C (room temperature). Subsequently, 200 μg of RTX was added to the tube and reacted for 10 min at room temperature. After labeling, an instant thin layer chromatography (solvent: 100% C_3_H_6_O) test showed that the radiochemical purity of ^131^I-RTX was > 95%. The immunoreactivity of ^131^I-RTX was determined as 87.7% by using a cell-binding assay and the specific activity was 86.2 ± 11.8 MBq/mg.

### 2.7. Radioimmunotherapy and Tumor Growth Delay

When the tumor volume in Raji-bearing mice reached ~200 mm^3^, the mice were randomly divided into five groups (*n* = 5–6 per group). Each group was treated with a single dose of PBS, ATV (12 μg/day in PBS), ^131^I-RTX (150 μg, 12.95 MBq), and ^131^I-RTX (150 μg, 12.95 MBq) plus ATV (12 μg/day in PBS).

### 2.8. SPECT/CT Image of ^131^I-Rituximab

All SPECT scans in this study were performed by using a Mediso nanoSPECT/CT scanner (Mediso, Budapest, Hungary). When the tumor size reached ~ 200 mm^3^, ATV (12 μg/day in PBS) was orally administered daily for a total of 10 days; PBS was administered to the control group. ^131^I-RTX (150 μg, 12.1–14.6 MBq/200 μL) was intravenously injected after 5 days of administration of ATV or PBS. SPECT data were obtained at 2, 24, 48, and 72 h after the injection of ^131^I-RTX. 

### 2.9. Autoradiography

Immediately after SPECT/CT scanning, the tumor tissues were isolated and frozen in an optimal cutting temperature (OCT) compound. After decaying for 48 h, the frozen tumors were sectioned at 20 μm thickness by using a cryostat microtome and exposed on an imaging plate for 24 h. The plates were scanned with BAS-5000 (Fujifilm Life science Co., Japan). The intensities of tumor uptake were quantified as units of photostimulated luminescence per square millimeter (PSL/μm^2^) computed by using Multi Gauge software (version 3.0; Fujifilm, Los Altos, CA, USA).

### 2.10. In Vivo Optical Imaging of Orthotopic Models

Luciferase-expressing Raji cells (1 × 10^6^) were injected into the tail vein of NOD/SCID mice for the establishment of orthotopic model. Seven days after cell injection, ^131^I-RTX (150 μg, 12.1–14.6 MBq/ 200 μL) was intravenously injected and ATV (12 μg/day in PBS) was orally administered daily for a total of 5 days; PBS was administered to the control group. For *in vivo* optical imaging, scans using IVIS Spectrum (Perkinelmer, Waltham, MA, USA) were performed 14 days after the cell injection. Bioluminescence images were acquired 6 min post-injection. Depending on bioluminescence intensity, the images were collected under various conditions with exposure times ranging from 1 to 60 s, binning from 4 to 16, and a 13.5-field of view. The total number of photons emitted per second was quantified using Living Image analysis software (v 4.5.1, Perkinelmer, Waltham, MA, USA).

### 2.11. In Vivo Tumor Model and Administration of ATV and miR-346 Mimic

Therapy was started when the tumor volume reached ~200 mm^3^. The tumor-bearing mice were randomly assigned to four groups (*n* = 5 each): (1) control mice receiving intraperitoneal (i.p.) injection of 100 μL PBS and intratumoral injection of 5 μg miRNA negative mimic; (2) mice receiving i.p. intratumoral injection of 5 μg has-miR-346 mimic; (3) mice receiving orally administered ATV (12 μg/day in PBS) and intratumoral injection of 5 μg has-miR-346 mimic; (4) mice receiving ATV (12 μg/day in PBS), intratumoral injection of 5 μg has-miR-346 mimic, and intratumoral injection of 5 μg has-miR-346 inhibitor. For in vivo administration of miRNA mimic, the negative control or miR-346 mimic was complexed with in vivo-JetPEI at an N/P ratio of 6 in 5% glucose solution (a total of 20 μL) for intratumoral injection. The mice were treated twice a week.

### 2.12. Immunocytochemistry of γH2AX

The cells were cultured with 5 μM ATV in 6-well plates for 2 days and then irradiated. At 3 h after 8 Gy of irradiation, the cells were fixed in 3.7% formaldehyde, blocked with 1% BSA/0.2% Triton-X-100 diluted in PBS, and stained with anti-γH2AX antibody (Abcam, Cambridge, UK) followed by a secondary antibody (anti-rabbit IgG-TRITC, Sigma, St. louis, MO, USA). The cells were then washed, loaded into a pre-assembled cytospin cuvette, and centrifuged at. 1000 rpm for 5 min in Shadon Cytospin 3 (Thermo Scientific, Eugene, OR, USA) to attach Raji cells. The cells were cover-slipped with mounting medium containing a 40, 6-diamidino-2-phenylindole dihydrochloride (DAPI, Invitrogen, Carlsbad, CA, USA) solution (Vector Laboratories, Inc., Burlingame, CA, USA). All groups were then assessed for γH2AX by using confocal microscopy (LSM 710, Carl Zeiss, Germany) with filters specific for DAPI (excitation/emission: 359/461 nm) and TRITC (excitation/emission: 555/580 nm). 

### 2.13. Whole Code Set Heat Map and Cluster Analysis

Unsupervised hierarchical analysis of the genes from control (*n* = 3) and ATV-treated mice (*n* = 3) tumor tissues was performed using NanoString nCounter gene expression assay. Z score corrected expression data were presented. Lower signal of expression was indicated in blue and higher signal in red. Two large clusters, cluster 1 (439 genes) and cluster 2 (279 genes), were identified (shown as pink and sky-blue). The top 10 gene ontology (GO) terms associated with cluster 1 (pink box) and cluster 2 (sky-blue boxes) from the unsupervised hierarchical cluster analysis were presented. 

### 2.14. NanoString nCounter Preparation, Operation, and Analysis

Seven minutes prior to euthanization and tumor removal, the total RNA was isolated from the tumor tissue by using QIAGEN Allprep kit (QIAGEN, Hilden, Germany). NanoString nCounter assay was performed using 300 ng of purified RNA from control and ATV-treated mice samples. The hybridization reaction was performed according to the manufacturer’s instruction, and the sample was incubated at 65 ℃ for at least 12 h before direct processing. After the experimental process and NanoString nCounter digital reading, the counts for all RNAs were extracted, normalized, and analyzed using a NanoString nSolver software and in-house MATLAB (Mathworks, MA, USA) code. The genes with a *p*-value < 0.05 were selected. For normalization, we used nCounter-nSolver analysis software (version 4.0, nanoString, Seattle, WA, USA), which included normalization process using housekeeping gene/positive control/negative control. 

### 2.15. Bioinformatics Analyses

The cluster genes were analyzed using the DAVID web toolkit (http://david.avcc.ncifcrf.goc) to assign GO categories and plotted by homemade MATLAB code, following the DAVID manual. To interrogate the interactions between our interesting cluster gene set and pathways, we used STRING according to the manual (Search Tool for Recurring Instances of Neighbouring Genes).

### 2.16. Cell Viability Assay and Cell Lysate Analysis

To examine the cytotoxicity induced by ATV and RTX in various lymphoma cells, we treated Raji, Jeko-1, Mino (mantle cell lymphoma), and U2932 cells with ATV for 2 days and RTX for 1 h in. To determine the concentration of ATV for viability of Raji cells, we treated the cells with various concentrations (0, 0.5, 1, 2, 5, 10, 20, and 50 μM) of ATV for 48 h. Cell viability was measured at an emission wavelength of 590 nm using a microplate reader. Next, to examine the cytotoxicity induced by ATV and RTX in various lymphoma cells, we treated the cells with ATV for 2 days and RTX for 1 h. Among variable doses, 10 μM showed effective cytotoxicity, corresponding to an inhibitory concentration of 25% at 48 h exposure.

Next, cytotoxicity induced by poly (ADP-ribose) polymerase-1, which is a unique characteristic of apoptotic cell death, was proven by immunoblotting of cell lysates using the indicated antibodies.

### 2.17. Western Blotting

The cells were subsequently lysed with RIPA buffer (Thermo Scientific, Eugene, OR, USA), and proteins were separated by sodium-polyacrylamide gel electrophoresis and transferred to nitrocellulose membranes. The membranes were blocked with 1% (v/v) non-fat dried milk in Tris-buffered saline with 0.05% Tween 20 and incubated with the required antibodies. Primary antibodies were used at a 1:1000 dilution (5% bovine serum albumin) and secondary antibodies at a 1:5000 dilution (5% skim milk). Immunoreactive protein bands were visualized via enhanced chemiluminescence (Amersham Biosciences, Freiburg, Germany) and scanned.

### 2.18. Reagents and Antibodies

The miR-346 mimics, control mimics, miR-346 inhibitors, and control inhibitors were all purchased from Bioneer (Daejeon, Korea). In vivo-jetPEI^®^in vivo DNA and siRNA delivery reagent was purchased from Polyplus-transfection^®^ SA (Polyplus, New York, NY) and lipofectamine (Thermo Fishers Scientific, Eugene, OR, USA). The primary antibodies used for western blot analyses were obtained as follows: Anti-HIF1alpha (BD Transduction Laboratories, San Jose, CA, USA); anti-VEGF, anti-cyclin B, anti-β-actin (Santa Cruz Biotechnology, Dallas, TX, USA); anti-cleaved PARP, anti-caspase-3, anti-p-cdc2 (Cell Signaling Technology Danvers, MA, USA); and anti-γ- H2AX (Millipore, Billerica, MA, USA). ATV was purchased from Sigma-Aldrich (St. Louis, MO, USA).

### 2.19. Matrigel In Vitro Endothelial tube Formation Assay

Endothelial cell tube formation assays were performed on Matrigel-coated chamber slides and the results were photographed (Nikon Eclipse Ti microscope with DS-Fi1 camera) at ×40 magnification. Tube formation was quantified by counting the number of connected cells in randomly selected fields at ×400 magnification and dividing that number by the total number of cells in the same field.

### 2.20. Quantitative RT-PCR Analysis

To verify the array results, quantitative RT-PCR (qRT-PCR) analysis was performed by using total RNA obtained from Raji cells treated with different concentrations of ATV. The total RNA was isolated by using RNeasy isolation Kit (QIAGEN, Hilden, Germany) and copy DNA (cDNA) was synthesized from 1 mg of RNA by using the miScript II RT kit (QIAGEN, Hilden, Germany) in accordance with the manufacturer’s instructions. qRT-PCR was performed by using miScript Primer Assays (QIAGEN, Hilden, Germany) and SYBR Green qPCR Master Mix (QIAGEN, Hilden, Germany) at 95 °C for 3 min and 40 cycles of 95 °C for 15 s and 62 °C for 30 s on. 7500 Real time PCR System (Applied Biosystems, Waltham, Massachusetts, USA). Each miRNA expression was represented as a fold-change relative to the expression of small RNA 5S rRNA, which was used as an internal control of qRT-PCR. The difference in miRNA expression was determined as a fold change using the 2^−ΔΔCt^ method.

### 2.21. Measurement of HIF-1α Subunit and VEGF Production by Enzyme-Linked Immunosorbent Assay

Intracellular HIF-1α and VEGF activity were assayed using the SimpleStep enzyme-linked immunosorbent assay (ELISA) kit (Abcam, Cambridge, UK) according to manufacturer’s instructions with plates assayed on a 96-well plate reader at 450 nm.

### 2.22. Microarray and Data Analysis

Using 250 ng of total RNA, the labeling process was initiated with poly-A tailing of each RNA strand with poly-A polymerase, followed by the ligation of biotin-labeled 3DNA dendrimer. Biotinylated RNA strands were hybridized at 48 °C for 18 h on an Affymetrix GeneChip miRNA 4.0 Array (Affymetrix, Hatfield, PA, USA). The GeneChip miRNA 4.0 Array was washed and stained in Affymetrix Fluidics Station 450. The fluorescence signals amplified by the branched structure of the 3DNA dendrimer were scanned by using an Affymetrix GeneChip Scanner. 3000 7G. The arrays were analyzed by using an Agilent scanner and the associated software. miRNA expression levels were calculated by using Expression Console 1.4 (Affymetrix, Hatfield, PA, USA). The relative signal intensities for each miRNA were generated by using the Robust Multi-Array Average algorithm. The target predictions were computed and analyzed by using TargetScan and microRNA.org databases.

### 2.23. miRNA and Transient Transfection

The cells were transiently transfected with 60 nM control or miR-346 mimics, or with 120 nM control or miR-346 inhibitors, using X-treme GENE siRNA Transfection Reagent (Roche, Indianapolis, IN, USA). We used miRNA scramble as a control. The sequence of miRNA-346 mimic was 3′ UCUCCGUCCGUACGCCCGUCUGU 5′. For irradiation analysis, at 2 days after transfection of the miRNA inhibitor and treatment with ATV, the cells were exposed to various doses of ionizing radiation.

### 2.24. Luciferase Assay

The cells were seeded into a 24-well plate and HIF1 3′UTR reporter constructs were co-transfected with miR-346 and pRL-CMV-Renilla internal control plasmid (Promega, Madison, WI, USA) using Lipofectamine. 2000. The luciferase activity was determined by the Dual-Glo^TM^ Luciferase assay system (Promega, Madison, WI, USA).

### 2.25. Irradiation

Raji cells were administered with/without 5 μM ATV and incubated at 37 ℃ for 2 days. Then, Raji cells were exposed to various doses of ionizing radiation from a 137Cs γ-ray source in Exposure Instrument Cammacell-40 (Atomic Energy of Canada, Ltd., Canada) at a dose rate of 3.81 Gy/min.

### 2.26. Three-dimensional (3D) Culture System

The cells were seeded in 96-well plates at a density of 1 × 10^4^ cells/well. In the 3D culture model, 96-well plates were pre-coated with Matrigel as a basement membrane by adding 40 µL of Matrigel to each well followed by incubation at 37 °C for 30 min. The cells were plated onto the gel in appropriate medium, and the wells were photographed after 10 days.

### 2.27. Neutral Comet Assay

To detect double-strand breaks (DSBs), a neutral comet (single-cell gel electrophoresis) assay was performed according to the manufacturer’s instructions (Trevigen, Gaithersburg, MD, USA). The cells were plated in 100 mm tissue culture dishes at 1 × 10^6^ cells/dish and incubated overnight. After ATV or miR-346 inhibitor exposure for 24 h, the cells were irradiated and incubated for 24 h. The cells were then immediately lysed at 4 °C for 1 h in lysis buffer (2.5 M NaCl, 100 mM ethylenediamine tetraacetic acid, 10 mM Tris-HCl, 1% N-lauroylsarcosine, 1% Triton X-100, 10% DMSO, pH 10.0) and subjected to neutral electrophoresis buffer at 4 °C. To detect DNA, the slides were stained with ethidium bromide and examined for fluorescence emission using a 515–560 nm excitation filter and 590 nm barrier filter. DNA damage was quantified through computer-assisted image analysis (Komet analysis software, ver. 3.1; Kinetic Imaging, Liverpool, UK) to integrate fluorescence intensity.

### 2.28. Invasion Assay

The invasive ability was measured *in vitro* using transwell chambers, according to the manufacturer’s protocol. Briefly, cells were seeded onto the membrane of the upper chamber of the transwell at a concentration of 4 × 10^5^ cells/mL in 150 μL of medium and left untreated or treated with ATV, radiation, miR-346 inhibitor, or a combination of both for 24 h. The medium in the upper chamber was serum-free, while the medium in the lower chamber contained 10% FBS as a source of chemo-attractants. The cells that passed through the Matrigel-coated membrane were stained with Cell Stain Solution containing crystal violet supplied in the transwell invasion assay (Chemicon, Millipore, Billerica, GA, USA) and photographed after 24 h of incubation.

### 2.29. Dosimetry

The radiation dose per unit of administered activity (mSv/MBq), effective dose in organ, and absorbed dose for the tumor region on the ^131^I-RTX SPECT in mice were calculated by using OLINDA/EXM software (OLINDA; Vanderbilt University, Nashville, TN, USA). To calculate the absorbed dose in the tumor, the sphere model of OLINDA was used. The tumor volume was calculated from ^131^I-RTX SPECT data with multiple slices of ROIs. The ROIs were delineated in the heart, stomach, intestine, liver, spleen, kidney, bladder, lung, bone, muscle, and tumor regions. After the delineation of ROIs on X-ray CT, ROIs were copied to the ^131^I-RTX SPECT data. Time activity curves (TACs) were obtained for each organ. 

### 2.30. Ethics Approval and Consent to Participate

All experiments were performed under a protocol approved by IACUC (KIRAMS 2013-0099, KIRAMS 2015-0026, KIRAMS 2016-0019) of the Korea Institute of Radiological and Medical Sciences (KIRAMS) and by IACUC (2017-106) of Osong Medical Innovation Foundation.

### 2.31. Statistical Analysis

The data were presented as the mean ± SD and calculated using Student’s *t* test. Statistical significance was accepted for *p* values of < 0.05 and 0.005.

## 3. Results

### 3.1. HIF-1α Expression in Raji Cell Line was Higher than that in Other Burkitt Lymphoma Cell Lines DLBCL or Leukemia

**Figure 1:**
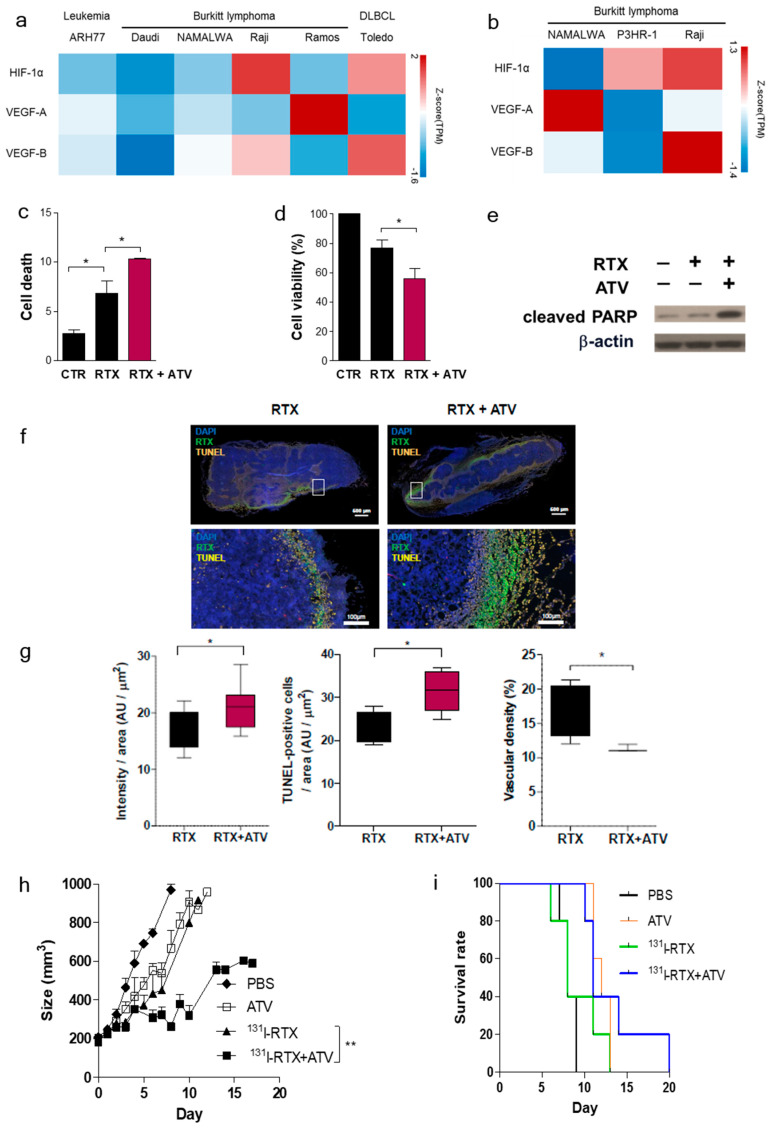
Atorvastatin (ATV) improved therapeutic effect of ^131^I-RTX in Raji cells. (**a**) Public RNAseq database, Expression ATLAS (EMBL-EBI, http://www.ebi.ac.uk/gxa). Expression ATLAS provided several immortalized B cell lines Project 6 of Open Targets—Epigenomes of Cell Lines. (**b**) RNAseq of 934 human cancer cell lines from the Cancer Cell Line Encyclopedia. (**c**, **d**) The apoptosis rate and cell viability were assessed by FACS analysis and trypan blue assay after atorvastatin (ATV) or ATV+ rituximab (RTX) treatment. (**e**) Apoptosis was confirmed by western blotting of cleaved PARP. Raji cell lysates are shown with the corresponding antibodies. (**f**) Fluorescence image of Alexa488- rituximab (RTX) was acquired using an In Cell Analyzer on a whole tumor tissue. Representative images of tumor tissue after treatment with Alexa488-RTX alone and combination with Alexa488-RTX and atorvastatin (ATV). Alexa488-rituximab (green), TUNEL-positive cell (yellow), and DAPI staining (blue). The lower row is an enlarged image of the white box in the upper row. (**g)** The accumulation of Alexa-488-RTX per tumor tissue area with or without ATV was quantified with TUNEL-positive cells per tumor tissue area, and the vascular density of RTX with or without ATV was quantified (*p < 0.05). (**h**) Tumor volume changes were measured in mice treated with PBS, ^131^I-RTX, ATV, and ATV plus ^131^I-RTX. The tumor volumes were calculated every other day for 20 days. (**p < 0.005). (**i**) Survival rates of each group.

To compare the expression level of HIF-1α, VEGFA, and VEGFB in leukemia, BL, and DLBCL, we used the public RNAseq database, Expression ATLAS (EMBL-EBI, http://www.ebi.ac.uk/gxa). Expression ATLAS provided several immortalized B cell lines Project 6 of Open Targets-Epigenomes of Cell Lines (Figure 1a) and RNAseq of 934 human cancer cell lines from the Cancer Cell Line Encyclopedia (Figure 1b) [18]. Transcripts per Million (TPM) value (cut off value: 0.5) was used. Z-score of TPM value was represented using a heatmap. The ATLAS RNAseq revealed that HIF-1α expression was higher in the Raji cell line than in leukemia or DLBCL. VEGFB expression level in Raji cell line was higher than that in other BL cell lines such as Daudi, NAMALWA, Ramos, and P3HR-1 (Figure 1a,b). Therefore, we selected Raji cells, which showed high resistance under a hypoxic environment.

### 3.2. Atorvastatin Inhibited Tumor Growth and Increased the Survival Rate of Lymphoma Model

ATV significantly decreased the viability of Raji cells compared with that of other lymphoma cell lines (Jeko-1, Mino, U2932) (Figure S2a). The viability of Raji cells was dose-dependently decreased by ATV (Figure S2b). Combination of RTX and ATV treatment significantly increased the percentage of early apoptotic cells and decreased the viability of Raji cells compared with the RTX only group (Figure 1c, d). PARP cleavage was enhanced in groups treated with ATV plus RTX compared with that in groups treated with RTX (Figure 1e). Figure 1f presents the enhanced accumulation of RTX after ATV treatment. The intensity of Alexa488-RTX and TUNEL-positive cells was 1.2-fold and 1.4-fold higher, respectively, during ATV treatment (Figure 1g; *p < 0.05). Vascular density significantly decreased in the ATV treated groups (Figure 1g; *p < 0.05). Tumor growth delay in ATV plus ^131^I-RTX group was significantly slower than that in ^131^I-RTX only groups (Figure 1h; **p < 0.005). 

The survival rates were also significantly greater in the combination group of ATV plus ^131^I-RTX than in the mice treated with ATV or ^131^I-RTX alone (Figure 1i and Figure S2c)

### 3.3. SPECT/CT and IVIS Images Showed that Atorvastatin Enhanced ^131^I-Rituximab Uptake in Raji Subcutaneous and Orthotopic Model

**Figure 2:**
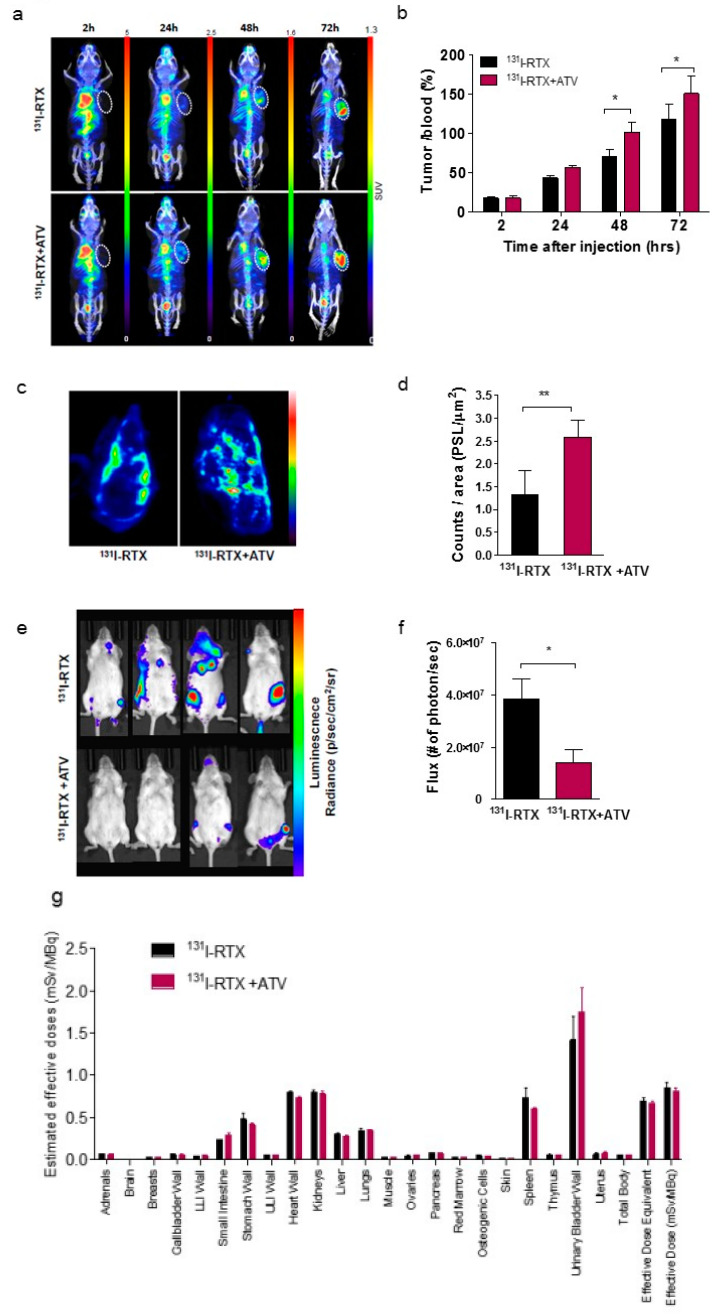
In vivo images of ^131^I-RTX after ATV treatment in Raji lymphoma tumor tissue (**a**) Representative SPECT/CT images of Raji-xenografted mice were acquired at 2, 24, 48, and 72 h after injection of ^131^I-RTX (upper row) and ATV plus ^131^I-RTX (lower row). White dotted circles indicate the tumor region. (**b**) The quantification of ^131^I-RTX accumulation in tumors is represented by the tumor to blood ratio at each time point (*p < 0.05). The data are the mean ± SD from five independent mice. (**c**) Autoradiography of ^131^I-RTX in Raji tumors was conducted after the acquisition of SPECT images (upper row). (**d**) The total accumulation of ^131^I-RTX per tumor tissue (**p < 0.005). The data are the mean ± SD from ten independent images. (**e**) IVIS images presented a lower signal for combination group of ATV plus ^131^I-RTX (lower row) than the ^131^I-RTX alone group (upper row) in Raji-luciferase cell xenografted orthotropic model. (**f**) Total number of photons per second was 1.42 × 107 for ^131^I-RTX and 3.84 × 107 for ^131^I-RTX + ATV (p* < 0.05). (**g**) The result of effective dose for ^131^I-RTX and ^131^I-RTX+ ATV.

Immuno-SPECT images demonstrated a higher uptake of ^131^I-RTX in tumors of the ATV-treated group than that of the PBS group (Figure 2a). The tumor to blood ratios (%) of ^131^I-RTX in ATV group were 1.4-fold and 1.2-fold higher than those in the PBS group at 48 and 72 h, respectively, after injection (Figure 2b; *p < 0.05). Autoradiography revealed a higher tumor uptake in the ATV-treated group than in the PBS group (Figure 2c,d). These results demonstrated that ATV increased the accumulation of ^131^I-RTX in tumors (Figure 2d; **p < 0.005). IVIS images presented a lower signal for the combination group of ATV plus ^131^I-RTX than for ^131^I-RTX alone group in Raji-luciferase cell xenografted orthotropic model (Figure 2e). The total number of photons per second was 3.84 × 10^7^ (for ^131^I-RTX) and 1.42 × 10^7^ (for ^131^I-RTX+ATV), respectively (Figure 2f; *p < 0.05). This finding indicates that the high uptake of ^131^I-RTX by ATV increased the cytotoxic effects of ^131^I-RTX in Raji-luciferase-orthotopic model. Although significant enhanced therapeutic effect was observed in the ATV plus ^131^I-RTX group compared with the ^131^I-RTX only group, no significant differences were observed between the groups in terms of effective dose of organ or tissue (Table 1 and Figure 2g), indicating that the deposited energy emitted from ^131^I to the general organ or tissue was within the normal range even after adding ATV as a combination drug. The absorbed doses of ^131^I-RTX in the tumor were 352.40 ± 61.48 and 386.00 ± 69.80 mGy/MBq for the PBS and ATV-treated groups, respectively. 

### 3.4. Atorvastatin Suppressed HIF-1α and VEGF, Leading to Reduced Tumor Angiogenic Activity in Atorvastatin-Treated Cells

**Figure 3:**
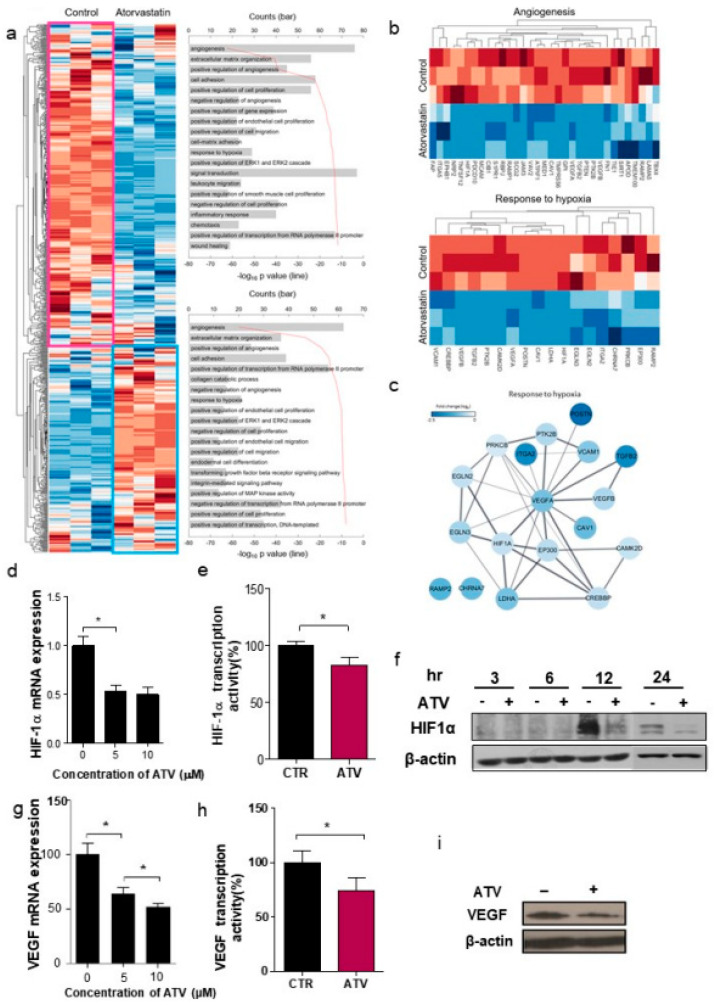
Atorvastatin suppresses HIF-1α and VEGF, leading to reduced tumor angiogenic activity in atorvastatin-treated cells. (**a**) NanoString analysis and unsupervised algorithms. Using 718 probe set with highest variable gene expression, two clusters of co-expressed genes were defined using hierarchical clustering. (**b**) Two clusters of co-expressed genes for angiogenesis and response to hypoxia. (**c**) String network analysis for response to hypoxia. HIF-1α, VEGF A, and VEGF B were highly correlated. (**d**) mRNA level of HIF-1α by qRT-PCR. qRT-PCR revealed that ATV attenuated the mRNA levels of HIF-1α in ATV-treated Raji cells. (**e**) Transcriptional activity of HIF-1α in ATV-treated cells. (**f**) HIF-1α protein level by western blotting. mRNA and protein level of HIF-1α, and transcriptional activity of HIF-1α also decreased in ATV-treated cells. The mRNA level of HIF-1α by qRT-PCR (**g**) The mRNA level of VEGF by qRT-PCR. qRT-PCR revealed that ATV attenuated the mRNA levels of VEGF in ATV-treated Raji cells. (**h**) Transcriptional activity of VEGF. (**i**) VEGF protein level by western blotting. The mRNA and protein level of VEGF, and transcriptional activity of VEGF were also decreased in ATV-treated cells.

We examined the differences in gene expression between the control and ATV-treated lymphoma tissues isolated from Raji xenograft tumors by NanoString analysis and unsupervised algorithms. Using 718 probe set with highest variable gene expression, two clusters of co-expressed genes were defined using hierarchical clustering (Figure 3a). Figure 3b presents two clusters of co-expressed genes for angiogenesis and response to hypoxia. Figure 3c depicts the string network analysis for response to hypoxia. We found that HIF-1α, VEGF A, and VEGF B were highly correlated (Figure 3c). 

We confirmed that ATV attenuated mRNA levels of HIF-1α in ATV-treated Raji cells by using qRT-PCR (Figure 3d). ATV-treated cells presented decreased transcriptional activity, as determined by western blotting and ELISA assay (Figure 3e; *p < 0.05). In hypoxia-induced Raji cells (hypoxia time: 9 h), the protein expression of HIF-1α was inhibited by atorvastatin for 12 and 24 h (Figure 3f). ATV inhibited the viability of Raji cells; however, hypoxia-induced Raji cells were less sensitive to ATV treatment than the control under hypoxic conditions. In addition, ATV treated groups under hypoxia were less sensitive than the groups under normoxia (Figure S3a; *p < 0.05). The mRNA, protein level, and transcriptional activity of VEGF also decreased in ATV-treated cells (Figure 3g–i; *p < 0.05). The dysfunction in tube formation was substantially reduced after ATV treatment (Figure S3b).

### 3.5. miRNA-346 Suppresses HIF-1α and VEGF Leading to Reduced Tumor Angiogenic Activity in Atorvastatin-Treated Cells

**Figure 4:**
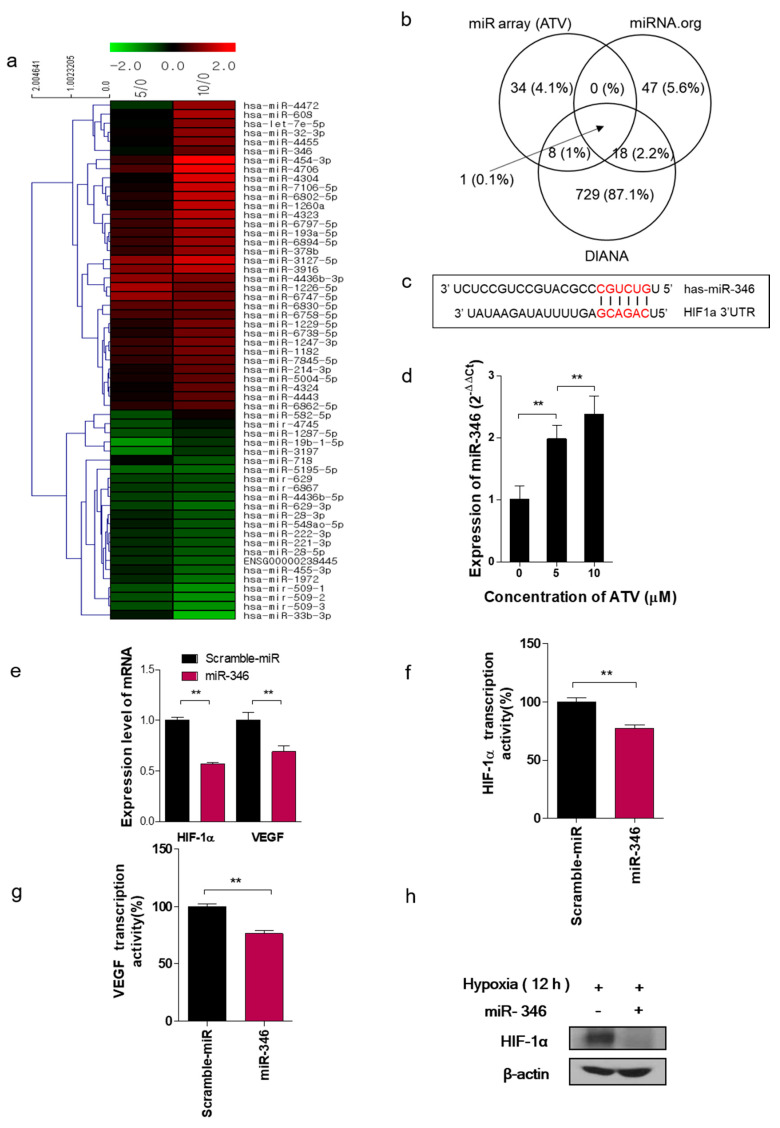
Target relationship between miR-346 and HIF-1a in the Raji cells. (**a**) Hierarchical clustering of miRNA expression reveals a descending ordered list of significant miRNA clusters in the untreated control cells. (**b**) Venn diagram presenting the number of putative miRNA candidates by two prediction programs and microRNA microarray. (**c**) The sequence alignment of miR-346 with the 3′UTR of HIF-1α gene was searched in the miRNA database. (**d**) The relative expression of miR-346 in Raji cells was analyzed according to the concentration of ATV via qRT-PCR (**p < 0.005). (**e**) The mRNA expression of HIF-1α and VEGF was measured by using qRT-PCR after miR-346 treatment in Raji cells. (**f**) The level of HIF-1α determined by ELISA after miR-346 transfection (**p < 0.005). (**g**) The level of VEGF determined by ELISA after miR-346 transfection (**p < 0.005). (**h**) The protein expression of HIF-1α determined by western blotting in hypoxia-induced-Raji cells.

The expression levels of 22 miRNAs were significantly upregulated, whereas those of 16 miRNAs were downregulated in ATV-treated Raji cells based on the miRNA array (Figure 4a). We employed two miRNA target predicting websites (miRNA.org and targetscan) and miRNA microarray analysis to predict the targets of HIF-1α. In total, 178 microRNAs were identified by all the three bioinformatics approaches; among these, a potent microRNA (miR-346) was identified equally at three sites (Figure 4b). We analyzed the 3′ UTR sequences of HIF-1α as well as the mature chain sequence of miR-346 and found that the “seed region” of the miR-346 mature chain was fully complementary to and thus could potentially bind to the 3′ UTR sequences of HIF-1α (Figure 4c). 

To test our hypothesis, we employed miRNA mimics to specifically overexpress the endogenous expression of miR-346 in Raji cells. The expression of miR-346, measured by qRT-PCR using a specific primer set, gradually increased with ATV concentration in Raji cells (Figure 4d; **p < 0.005). Cell viability significantly decreased after the cells were transfected with miR-346 mimics, and the level of decrease was prominent in ATV-treated cells (Figure S4a; *p < 0.05). The relative firefly luciferase activities of CMV/Fluc/HIF1a-3′UTR normalized with Renilla luciferase were measured 48 h post-transfection; the results are plotted as a percentage change over respective controls (Figure S4b; **p < 0.005). The expression of HIF-1α and VEGF significantly decreased after the cells were transfected with miR-346 mimics at the mRNA levels (Figure 4e). We confirmed that HIF-1α and VEGF transcription activities were inhibited in miR-346-treated cells when compared with the controls (Figure 4f,g; **p < 0.005). The protein level of HIF-1α decreased after ATV treatment (Figure 4h). The dysfunction of tube formation was significantly decreased after treatment with miR-346 in hypoxic condition (Figure S4c). Our findings suggest that miR-346 augments HIF-1α and inhibits the tumor angiogenic activity in ATV-treated cells. Thus, we hypothesized that miR-346 might play a pivotal role in angiogenesis by directly targeting HIF-1α to ATV and negatively regulating its expression. 

To determine whether the overexpression of HIF-1α counteracts the effect of miR-346 in lymphoma cells, we co-transfected miR-346 mimic or mimic control with or without HIF-1α overexpression vector into Raji cells. As assumed, miR-346 significantly decreased the levels of HIF-1α and VEGF, whereas co-expressing HIF-1α^Δ3′UTR^ restored the transcription activity of HIF-1α and VEGF (Figure S4d,e; **p < 0.005, * *p <* 0.05). In addition, the decreased cell viability induced by miR-346 was restored by HIF-1α^Δ3′UTR^ overexpression (Figure S4f). 

### 3.6. Overexpression of miR-346-Inhibitor Restores the Inhibitory Effect of miR-346 in Raji Cells

To investigate whether miR-346 could regulate HIF-1α under endogenous conditions, we blocked the function of miR-346 using antisense oligonucleotides (miR-346-inhibitor). Raji cells were transfected with various concentrations (0, 2, 4, and 8 μM) of miR-346-inhibitor (Figure S5a; **p < 0.005).

**Figure 5:**
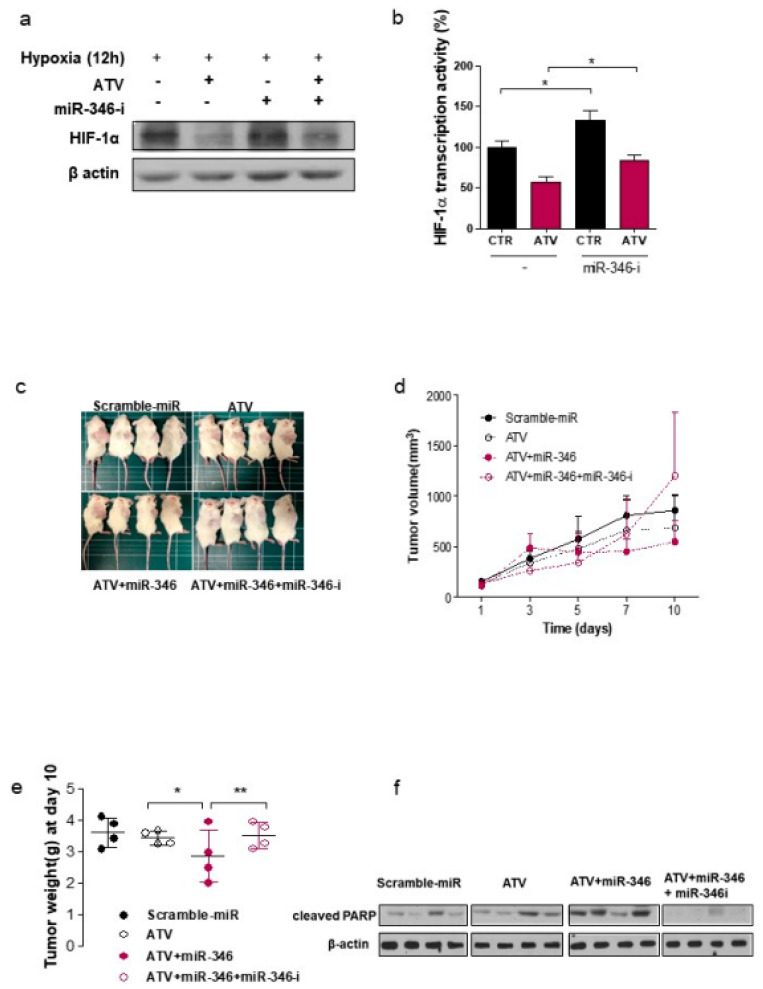

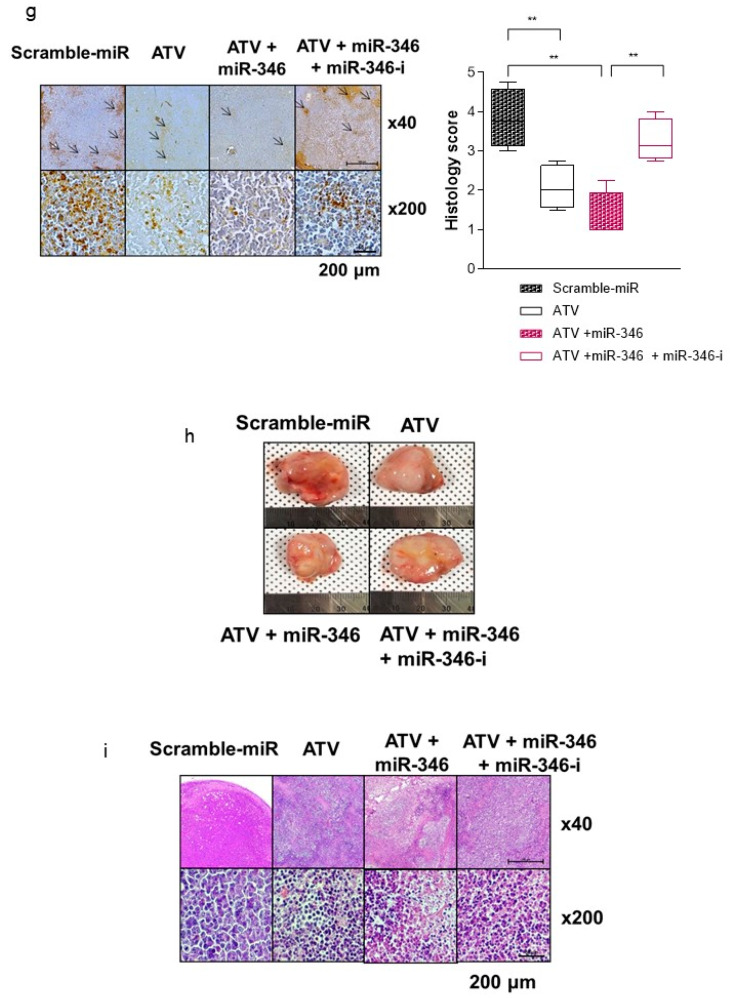
Target relationship between miR-346 and HIF-1a in Raji cells. (**a**) The cell lysates were immunoblotted with the indicated antibodies after miR-346 inhibitor treatment (*p < 0.05). (**b**) Cells were treated with ATV and miR-346 inhibitor for 24 h, and ELISA was performed to quantify the level of HIF-1α transcription activity. (**c**) Representative images of Raji tumor-bearing mice after injection of each treatment. (**d**) *In vivo* tumor growth delay of Raji xenografts intratumorally-treated with ATV, ATV+miR-346, ATV+miR-346+miR-346 inhibitor. (**e**) Tumors were excised and weighed at the end of the experiment. (*p < 0.05; **p < 0.01). (**f**) Western blots of cleaved PARP protein levels in Raji xenografts. (**g)** HIF-1α immunohistochemical stain levels in mice receiving each treatment and corresponding result of IHC scoring. (**h**) Representative images of tumors isolated from each treated mice, *n* = 4. (**i**) Hematoxylin and eosin (H&E) staining was examined by immunohistochemistry.

In hypoxia-induced Raji cells, the protein expression of HIF-1α, which could have been inhibited by ATV, was restored by miR-346 inhibitor (Figure 5a). To further confirm the offset on miR-346 inhibition by HIF-1α overexpression, we examined the tube forming ability of HUVECs. Tubular formation was reduced by 75% in HUVECs treated with miR-346 conditioned medium; it was restored in the presence of HIF-1α^Δ3′UTR^, indicating the essential role of HIF-1α in miR-346-suppressed tube formation (Figure S5b). All these effects were reversed by co-expressing a HIF-1α^Δ3′UTR^ construct and miR-346 inhibitor, emphasizing the direct target of miR-346 in HIF-1α regulation. miR-346 inhibitor increased the production of HIF-1α transcription activity with or without ATV (Figure 5b; *p < 0.05).

Based on the in vitro findings, we examined the effect of miR-346 in SCID mouse models of human lymphoma assigned to one of the four groups—control; ATV only; ATV plus miR-346; and ATV plus miR-346 plus miR-346 inhibitor—to explore whether this phenotype can be therapeutically exploited. The animal experiments showed that there was a tendency to delay tumor growth, but the difference was not statistically significant. However, in view of the results of cell-level experiments and tumor weight (Figure 5e), we believe that ATV may delay tumor growth. Notably, when ATV was combined with miR-346 mimics, a significant reduction was observed in tumor growth when compared with control mice or ATV-treated mice. The mice treated with miR-346 inhibitor recovered the inhibitory effect in terms of tumor weight (Figure 5c–e; **p < 0.01, *p < 0.05). To determine whether miR-346 mimics enhanced apoptosis in ATV-treated groups, we evaluated the apoptotic marker cleaved PARP by western blotting. The results revealed that apoptosis induction by ATV was increased by miR-346 mimic treatment (Figure 5f). The mRNA and immunohistochemistry (IHC) staining levels of HIF-1α were markedly reduced in Raji cells treated with ATV plus miR-346 compared with those in ATV alone treatment group; miR-346 inhibitor treatment recovered these events (Figure 5g). We also performed IHC scoring [19] for the quantification of HIF-1α levels (Figure 5g). According to the result of IHC scoring, the level of HIF-1 α decreased after ATV (**p < 0.005) and ATV + miR-346 (**p < 0.005) treatments. However, the level of HIF-1 α increased after treatment with miR-346 inhibitor (**p < 0.005) (Figure 5g). Figure 5h shows the representative images of tumors isolated from each treated mice. Control tumors exhibited a homogeneous distribution of viable cells, and the sections of ATV+miR-346-treated tumors presented much higher apoptotic evidence of cell shrinkage, nuclear condensation, and fragmentation via H&E staining than the ATV group. Nevertheless, these events were inhibited in ATV+miR-346+miR-346-I groups (Figure 5i). Notably, miR-346 administration did not produce any significant behavioral changes or weight loss in the treated animals. No weight change in the spleen and lung was detected, indicating the absence of acute toxicity (Figure S5c). The above findings indicate that the therapeutic delivery of miR-346 led to the repression of HIF-1α in lymphoma and consequently, enhanced the tumor inhibitory effects of ATV. 

### 3.7. Biological Activity of Atorvastatin and Radiation in Raji Cells by Regulating miR-346

**Figure 6:**
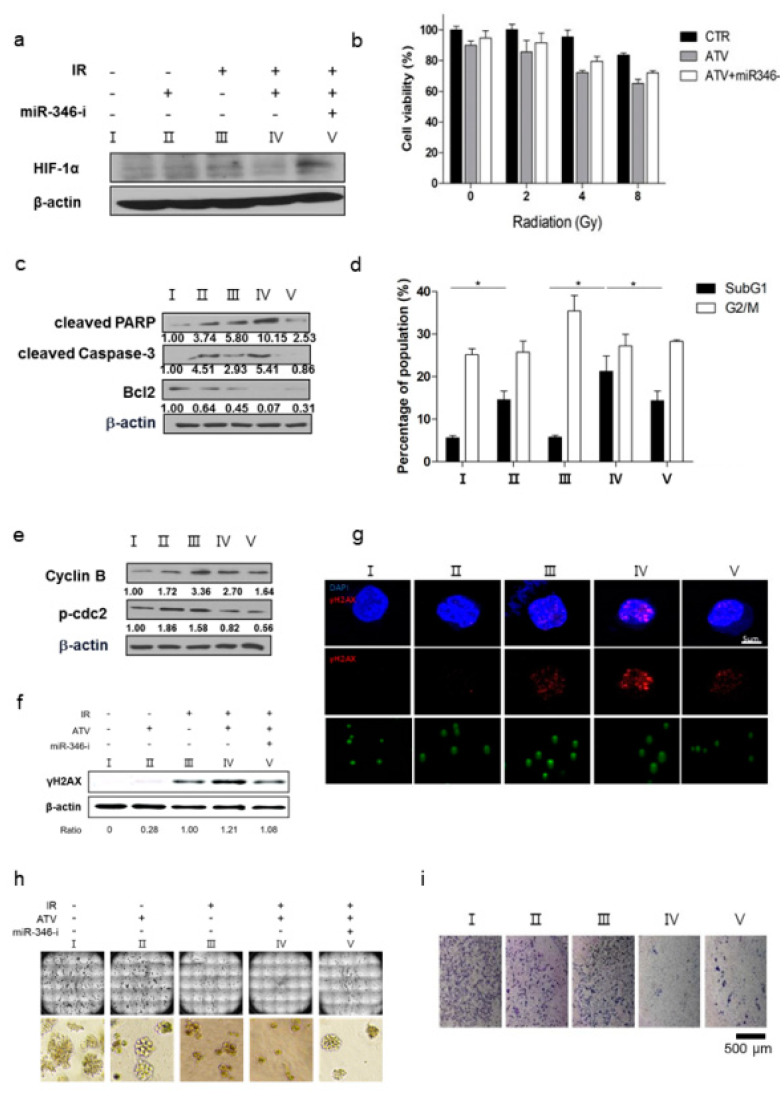
Atorvastatin and miR-346 regulated the radiosensitivity of Raji cells. (**a**) The cell lysates were immunoblotted with the indicated antibodies after atorvastatin and radiation and miR-346 inhibitor treatment (*p < 0.05). (**b**) The cell viability was measured by CCK8-assay in Raji cells treated with atorvastatin and/or miR-346-inhibitor under 2, 4, and 8 Gy irradiation. (**c**) Cell lysates were immunoblotted with the indicated antibodies. (**d**). Cell cycle analysis was conducted by PI-staining in each group at 24 h after IR (*p < 0.05). The data are the mean ± SD from three independent groups. (**e**). Cell lysates were immunoblotted with the indicated antibodies. Western blotting showed that radiation alone led to significant accumulation of cyclin B and p-CDC2, a key cell cycle regulator involved in the G2/M transition. (**f**) γH2AX expression was measured by western blotting in cell lysates of each group at 3 h after IR. (**g**) Immunofluorescence staining of γH2AX in each group and comet assay was imaged by confocal microscopy (blue; DAPI, red; γH2AX). (**h**) The formation of colonies in 3D culture was photographed by using an In Cell analyzer after various treatments. (**i**) Tumor cell invasion was assessed using Matrigel invasion assay.

To examine whether miR-346 was directly regulated by ATV in combination with radiation, we determined the protein level of HIF-1α after ATV and radiation in miR-346 inhibitor-treated cells. As presented in Figure 6a, the decreased level of HIF-1α by combination treatment was restored after miR-346 inhibitor treatment, suggesting that miR-346 targeted HIF-1α in the combination therapy. These results suggested that ATV could not only induce apoptosis in the control and hypoxia-induced cells, but also enhance radiosensitivity in Raji cells. Furthermore, we investigated the impact of ATV and miR-346 inhibitor on the radiosensitivity of Raji cells. The results revealed that the cell viability of ATV-treated group was significantly and dose-dependently decreased by irradiation (Figure 6b, *p < 0.05, **p < 0.005); however, a reversal of effect, i.e., increase in cell viability, was observed when the cells were treated with ATV and miR-346-inhibitor. These results suggested that ATV enhanced IR-induced apoptosis and miR-346 played a crucial role in ATV-enhanced apoptosis of Raji cells. Additionally, we observed increase in apoptosis after treatment with ATV under hypoxic condition. ATV increased apoptosis in both control and hypoxia-induced Raji cells compared with the controls. In addition, ATV increased irradiation-induced apoptosis in the control or hypoxia-induced Raji cells, when compared with irradiation alone (Figure S6b; *p < 0.05). Nonetheless, no differences were observed between ATV+IR and IR alone group, indicating that the cytotoxic effect of ATV was comparable to that of IR. These results were confirmed by using colony formation assays and 3D cultures (Figure 6h). We also examined whether ATV enhanced the radiation cytotoxicity resulting from further activation of caspase-3 and PARP fragmentation, the chief executioners of cell death, in Raji cells (Figure 6c). Our results revealed that caspase-3 activation and PARP cleavage was enhanced after ATV treatment in combination with irradiation compared with that observed in groups treated with ATV alone. Caspase-3 activation and PARP cleavage were blocked by treatment with miR-346-inhibitor

In addition, after treatment with ATV and IR, the most obvious changes in cell cycle distribution were increased G2/M phase cells in a single IR treatment. Conversely, in the case of additional treatment with miR-346-inhibitor, a 0.69-fold reduction in subG1 arrest was observed, but no significant change was observed in G2/M arrest when compared with combination treatment of ATV and IR (Figure 6d). Consistently, western blotting revealed that radiation alone led to significant accumulation of cyclin B and p-CDC2, a key cell cycle regulator involved in G2/M transition. Combination treatment with ATV and radiation decreased radiation-induced accumulation of cyclin B and p-CDC2. Consistent with the results of FACS analysis, the expression levels of these two proteins remained unaltered after miR-346-inhibitor treatment (Figure 6e). To examine the DNA damage caused by ATV and IR treatment, the formation of γH2AX was analyzed by western blotting and immunocytochemistry (ICC). Western blotting revealed a significant increase in γH2AX expression in a single IR treatment. Moreover, γH2AX expression was higher after combination treatment with ATV and IR than after a single IR treatment (Figure 6f); γH2AX expression was blocked by miR-346-inhibitor. The ICC results also revealed that γH2AX foci in the nucleus were in accordance with the western blotting results, colony assays, and comet assay (Figure 6f–h). These findings suggested that ATV enhanced the IR-induced DNA damage of Raji cells by regulating miR-346. To determine whether miRNA-346 affected the invasive activity of ATV and IR treatment, transwell chamber assay was performed in Raji cells 48 h after miRNA transfection. As presented in Figure 6i and Supplementary Figure 6a, miR-346 inhibitor treatment significantly recovered the inhibition of invasive activity of ATV- and IR-treated Raji cells compared to the untreated control. Collectively, these data suggested that ATV modulated radiosensitivity through miR-346. 

## 4. Discussion

In the present study, we reported for the first time that ATV improved the therapeutic effect of RIT using ^131^I-RTX. In addition, we proposed three hypotheses to elucidate the cause of the enhanced effect by ATV.

First, we investigated whether ATV improved the penetration of antibody into tumor by analyzing the fluorescence images of Alexa488-RTX and SPECT images of ^131^I-RTX (Figure 1f and Figure 2a). Statins have been demonstrated to induce apoptosis by regulating Akt, Erk, and p38 signaling through the suppression of mevalonate pathway in lymphoma cells [20]. We confirmed the increased apoptosis of ATV-treated lymphoma cells by using cell viability assay (Figure S2a) and upregulated expression of miR-1182 and miR-608, which was related to inhibition of tumor growth and induction of cell death [21,22], according to ATV concentration by microarray and qRT-PCR (Figure 5a and Figure S6c,d). The induction of cancer cell death enhanced intratumoral penetration via formation of void space in tumor tissues [23]. An increase in penetration of ^131^I-RTX resulted in increased absorption of the tumor dose, as presented by SPECT images, leading to DNA damage. DNA damage was proven by comet assay, western blotting of γH2AX, and colony assay (Figure 6f–h). Tumor absorbed dose was calculated using OLIDNA program [24]. The sphere model in OLINDA program provides the absorbed dose for the sphere mass in the range of 0.01–6000 g [24]. Based on the absorbed dose, tumors in the PBS group received 175.5 mGy irradiation and those in the ATV-treated group received 210.7 mGy irradiation. We presumed that ATV may induce 20% more DSBs because the number of lesions induced in the DNA of a cell by a dose of 1–2 Gy was approximately 40 DSBs in cell killing [25]. We speculated that the apoptosis increase by ATV resulted in increased penetration of ^131^I-RTX, which led to enhanced therapeutic effect of RIT in Raji cells. 

Second, we found that vascular formation decreased in vitro and in vivo in ATV-treated Raji cells (Figure 1g and Figure S3b). Additionally, we found that the gene expression related to angiogenesis, for example, VEGF, was reduced in ATV-treated Raji cells. VEGF signaling promotes the formation and branching of neovascularization in tumors, leads to rapid tumor growth, and facilitates metastatic potential [26]. Thus, we suggested that ATV induced anti-angiogenesis effects. 

Third, we confirmed the improvement of radiosensitivity by ATV by using cell viability assays, colony assays, cell cycle analysis, and γH2AX formation (Figure 6). When the same dose of radiation was irradiated, radiosensitive cells were subjected to more DNA damage than radioresistant cells because irradiation-induced DSBs led to apoptotic or mitotic death in cancer cells [27]. Therefore, the enhancement of radiosensitivity by ATV improved the therapeutic effect of RIT in Raji cells. However, ATV treatment as an option for combination therapy with ^131^I-RTX should be tailored according to the cell subtype. In the present study, we found that ATV was the most effective in Raji lymphoma cells based on the results of cell viability assay: ATV exhibited the highest cytotoxicity against Raji cells when compared with other cells such as MINO, U2932 (DLBCL), and Jeko-1 in (Figure S2a). This finding indicated that selection of ATV for combination therapy with ^131^I-RTX should be personalized according to the cell subtype. 

The enhanced uptake of ^131^I-RTX can be explained by the results of Tunel assay (Figure 1g). TUNEL assay showed that the proportion of apoptotic cells increased after treatment with ATV. The total uptake of ^131^I-RTX could be increased after reducing the interstitial pressure [28] due to apoptotic effect of ATV treatment. Similarly, enhanced uptake of mAb can be found after combinatorial treatment with chemotherapy drugs. After treatment of paclitaxel and HIFU, tumor accumulation and penetration of ^90^Y-B3 were enhanced [29,30]. Reduced interstitial pressure has been reported to be another reason for this enhanced uptake of mAb [29,30].

Combinatorial treatment approach for the enhancement of mAb could be applied during RIT in solid tumor. The barriers of tumor microenvironment, such as extracellular matrix, cell to cell junction, and high interstitial pressure, should be overcome during RIT in solid tumor [31,32]

In this study, we noted the changes in miRNA after ATV treatment to elucidate the mechanism of enhanced radiosensitivity in Raji cells. No report on the relationship between ATV and miRNAs has been published to date. In the present study, overexpression mimics of miR-346 in Raji cells abrogated the mRNA levels of the angiogenesis-related proteins HIF-1α and VEGF under hypoxia and the relative increase of HIF-1α protein levels in cells transfected with an miR-346 inhibitor [33], demonstrating that miR-346 targets the HIF-1α/VEGF axis (Figure 4e). Consistently, ATV attenuated VEGF expression at the protein level, reduced VEGF secretion, and reduced HIF-1α expression in Raji cells transfected with miR-346 mimics compared with the untreated control, indicating the anti-angiogenic property of miR-346. Moreover, we confirmed that miR-346 was upregulated by ATV in Raji cells as determined by miRNA microarray and qRT-PCR analysis. Furthermore, the overexpression of miR-346 synergistically reduced VEGF production by lymphoma cells, implying an important role of miR-346 in ATV-induced anti-angiogenic activity. In the mouse models, increased expression of miR-346 induced by ATV treatment promoted significant tumor growth delay compared with that in the control. These findings suggest that increasing the expression of miR-346 could be a potential therapeutic strategy. Furthermore, we identified that miR-346 inhibition negated the enhanced radiosensitivity induced by ATV via stimulation of IR-induced DNA damage, apoptosis, G2/M cell cycle arrest, and angiogenesis (Figure 6d). 

## 5. Conclusions

We demonstrated that upregulation of miR-346 induced by ATV in Raji cells increased RTX transport and radiosensitivity. Combination therapy with ATV could be a promising strategy for the treatment of hematological malignancies owing to its ability to improve radiosensitivity and anti-angiogenic property.

## Figures and Tables

**Table 1:** Estimated absorbed doses (mSv/MBq).

Target Organ	PBS		Atorvastatin	
	Mean	^*^SD	Mean	SD
Adrenals	6.35.E-02	2.84.E-03	5.98.E-02	1.88.E-03
Brain	3.56.E-03	3.26.E-04	3.32.E-03	2.91.E-04
Breasts	2.73.E-02	8.23.E-04	2.57.E-02	4.44.E-04
Gallbladder Wall	5.97.E-02	2.04.E-03	5.79.E-02	1.79.E-03
LLI Wall	3.90.E-02	1.08.E-02	4.62.E-02	8.12.E-03
Small Intestine	2.31.E-01	2.17.E-02	2.86.E-01	4.95.E-02
Stomach Wall	4.80.E-01	1.51.E-01	4.14.E-01	2.85.E-02
ULI Wall	4.79.E-02	4.70.E-03	5.32.E-02	2.62.E-03
Heart Wall	7.93.E-01	2.54.E-02	7.27.E-01	2.54.E-02
Kidneys	7.92.E-01	6.14.E-02	7.79.E-01	7.14.E-02
Liver	3.03.E-01	1.06.E-02	2.79.E-01	9.61.E-03
Lungs	3.41.E-01	4.11.E-02	3.49.E-01	9.45.E-03
Muscle	3.03.E-02	2.91.E-03	3.09.E-02	1.26.E-03
Ovaries	4.26.E-02	1.04.E-02	5.06.E-02	6.30.E-03
Pancreas	8.04.E-02	8.80.E-03	7.32.E-02	1.42.E-03
Red Marrow	2.82.E-02	2.01.E-03	2.84.E-02	5.22.E-04
Osteogenic Cells	4.64.E-02	2.74.E-03	4.42.E-02	9.65.E-04
Skin	1.27.E-02	1.09.E-03	1.25.E-02	3.13.E-04
Spleen	7.34.E-01	2.61.E-01	5.97.E-01	3.26.E-02
Thymus	5.81.E-02	1.90.E-03	5.41.E-02	1.20.E-03
Urinary Bladder Wall	1.41.E+00	6.31.E-01	1.74.E+00	6.47.E-01
Uterus	6.43.E-02	2.10.E-02	7.75.E-02	1.77.E-02
Total Body	5.57.E-02	3.44.E-03	5.50.E-02	6.34.E-04
Effective Dose Equivalent	6.95.E-01	9.47.E-02	6.73.E-01	3.08.E-02
Effective Dose (mSv/MBq)	8.81.E-01	1.38.E-01	8.29.E-01	7.19.E-02

^*^SD: standard deviation.

## Supplementary Materials

The following are available online at https://www.mdpi.com/2072-6694/12/5/1203/s1, Figure S1: Schematic of study design. RTX (anti CD20 antibody), ^131^I-RTX was used as a tool of targeted therapy, Figure S2: (a) After treatment with 10 μM ATV or 5 μg/mL RTX in Raji, Jeko-1, Mino, and U2932 lymphoma cells for 2 days, cell viability was determined by alamarBlue assay. (b) After treatment with various concentrations of ATV in Raji cells for 2 days, cell viability was determined by alamarBlue assay. The data were expressed as a percentage of the mean value of untreated control cells and the mean ± SD from three independent experiments. (c) Tumor volume of each mouse, Figure S3: (a) ATV inhibited the viability of Raji cells, but hypoxia-induced Raji cells were less sensitive to ATV than the control. (b) The dysfunction in tube formation was significantly decreased after ATV treatment, Figure S4: (a) Cell viability was determined after treatment with ATV and miR-346 transfection. The proliferation rate was measured by trypan blue exclusion method of cell viability. (b). The cells were co-transfected with either miR-346 or control mimics and 200 ng pGL3 reporter construct containing wild-type. The relative firefly luciferase activities normalized with Renilla luciferase were measured 48 h post-transfection, and the results are plotted as percentage change over respective controls (c). Tumor cell invasion and angiogenesis were assessed using Matrigel invasion assay and tube formation assay. Representative photomicrographs of in vitro tube formation. We co-transfected miR-346 mimic or mimic control with or without ATV treatment into Raji cells. (d) Transcriptional activity of HIF-1α (***p* < 0.005, **p* < 0.05). (e). Transcriptional activity of VEGF (***p* < 0.005, **p* < 0.05). (f) Cell viability: the decreased cell viability induced by miR-346 was restored by HIF-1αΔ3′UTR overexpression (***p* < 0.005, **p* < 0.05), Figure S5: (a) The relative expression of miR-346 in Raji cells was analyzed according to the concentration of miR-346 inhibitor by qRT-PCR (***p* < 0.005). (b) Representative photomicrographs of in vitro tube formation after each treatment. (c) No weight change in the spleen and lung was detected, clearly indicating absence of acute toxicity, Figure S6: (a) Relative cell tumor invasion (%) by Matrigel invasion assay. (b) Cell viability: control and hypoxia-induced Raji cells received irradiation (6 Gy), atorvastatin treatment, or irradiation (6 Gy) + ATV for 48 h. (c, d) Upregulated expression of miR-1182 and miR-608, which are related to inhibition of tumor growth and induction of cell death.
