# Supplementary material for: Inhibition of HIF-1α by Atorvastatin During 131I-RTX Therapy in Burkitt’s Lymphoma Model
Source: Cancers (Basel). 2020 May 11;12(5):1203. doi: 10.3390/cancers12051203 (PMC7281655; doi:10.3390/cancers12051203)
Supplement: Supplementary file 1 [file cancers-12-01203-s001.pdf]

cancers
MDPI

Article

Inhibition of HIF-1α by Atorvastatin during ^{131}I-RTX Therapy in Burkitt's Lymphoma Model

Eun-Ho Kim, Hae Young Ko, A Ram Yu, Hyeongi Kim, Javeria Zaheer, Hyun Ji Kang, Young-Cheol Lim, Kyung Deuk Cho, Hyun-Yoo Joo, Min Kyoung Kang, Jae Jun Lee, Seung-Sook Lee, Hye Jin Kang, Sang Moo Lim and Jin Su Kim

Supplementary Materials:

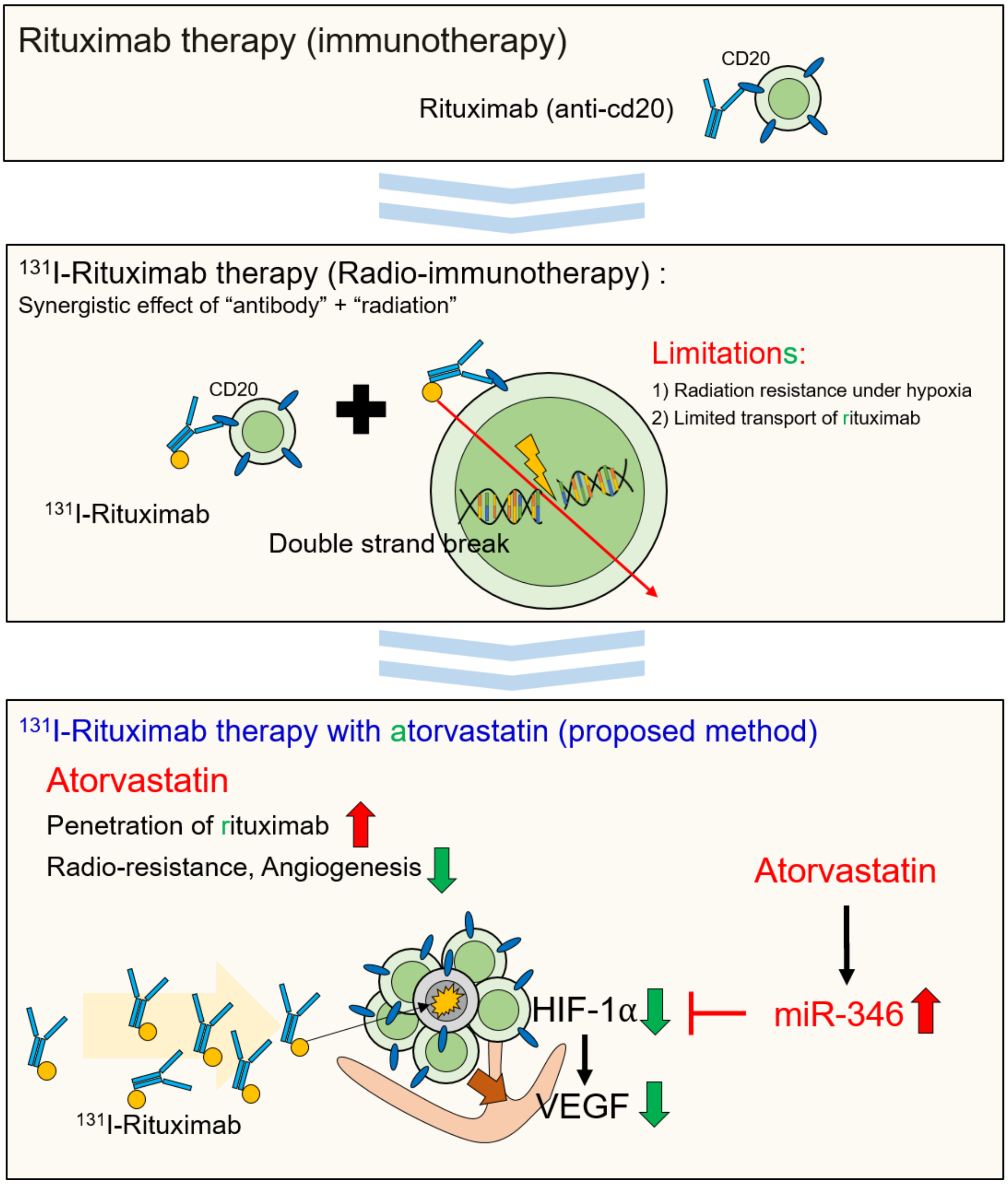

Figure S1. Schematic of study design. RTX (anti CD20 antibody), ^{131}I-RTX was used as a tool of targeted therapy. ^{131}I-RTX provided synergistic effect of antibody and DNA double strand break due to ^{131}I; however, there were several limitations during ^{131}I-RTX, indicating radiation resistance under hypoxia condition and limited transport of RTX. Therefore, in this present study, we proposed ATV combination study with ^{131}I-RTX. We confirmed that HIF-1α and VEGF were downregulated after ATV treatment.

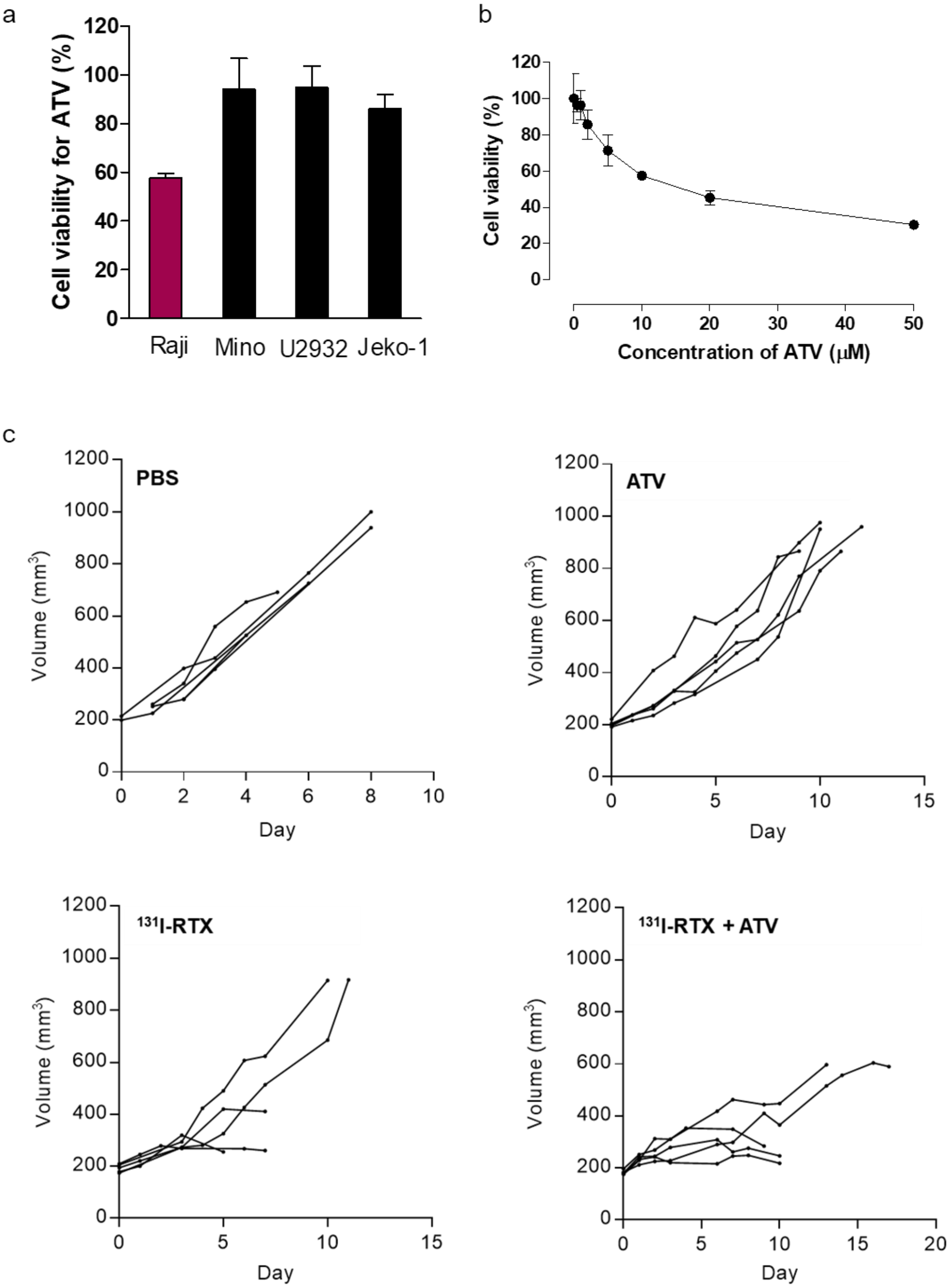

Figure S2. (**a**) After treatment with 10 μM ATV or 5 μg/mL RTX in Raji, Jeko-1, Mino, and U2932 lymphoma cells for 2 days, cell viability was determined by alamarBlue assay. (**b**) After treatment with various concentrations of ATV in Raji cells for 2 days, cell viability was determined by alamarBlue assay. The data were expressed as a percentage of the mean value of untreated control cells and the mean ± SD from three independent experiments. (**c**) Tumor volume of each mouse

a

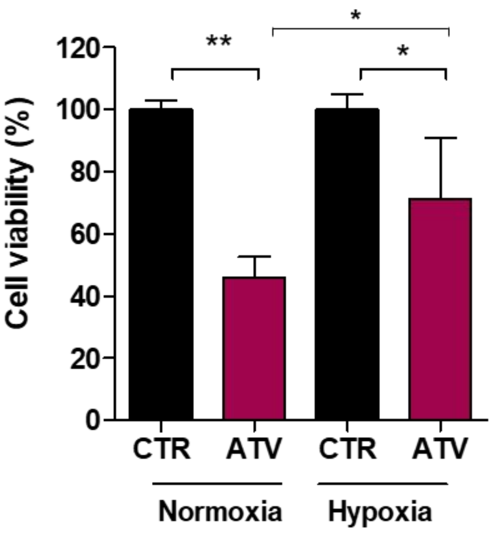

b

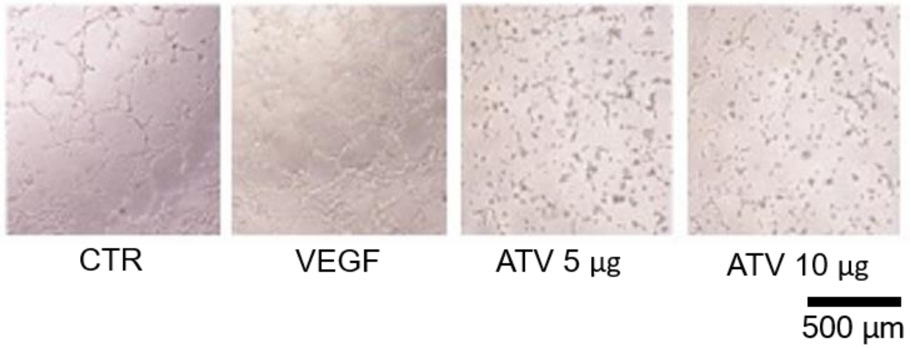

Figure S3. (**a**) ATV inhibited the viability of Raji cells, but hypoxia-induced Raji cells were less sensitive to ATV than the control. (**b**) The dysfunction in tube formation was significantly decreased after ATV treatment.

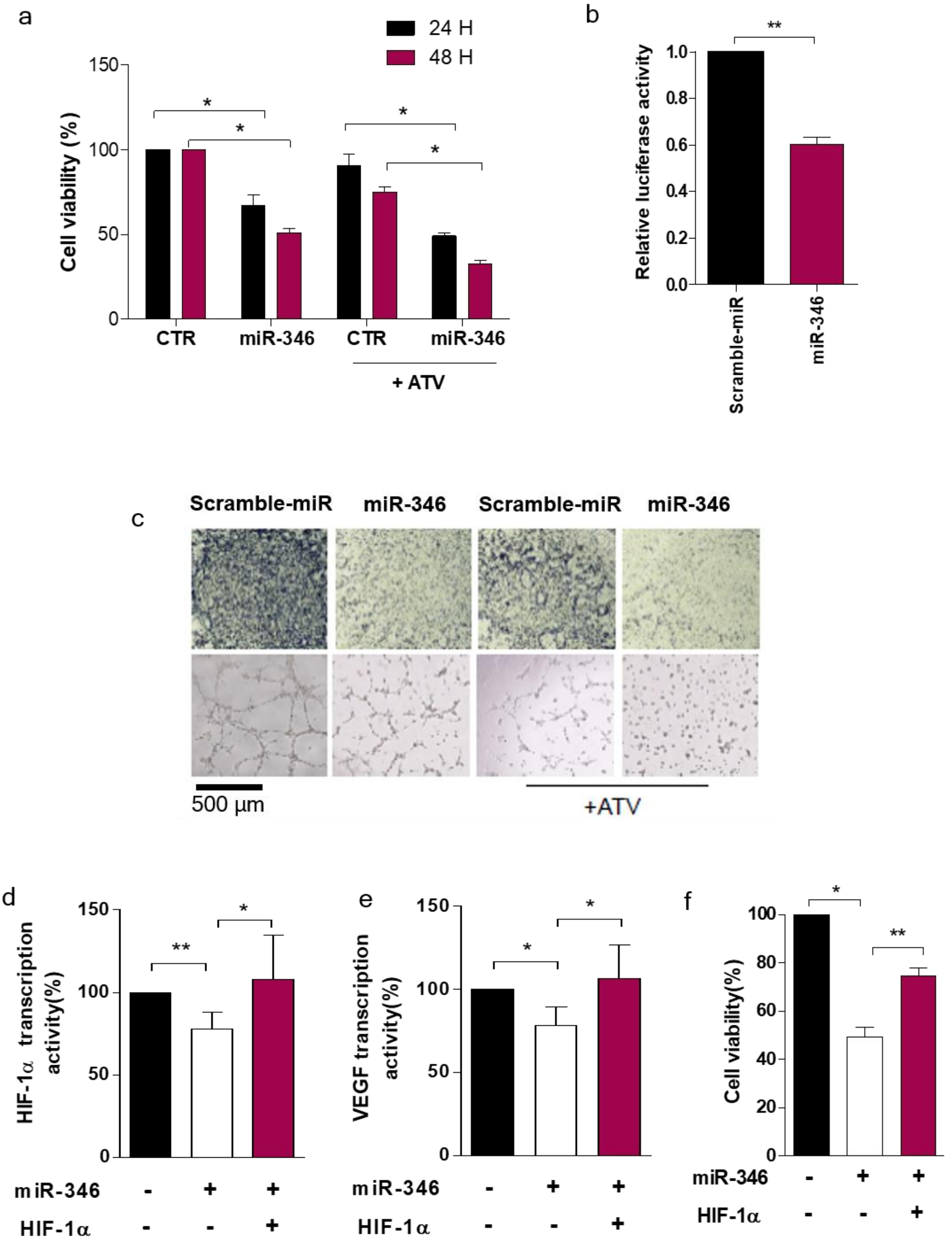

Figure S4. (**a**) Cell viability was determined after treatment with ATV and miR-346 transfection. The proliferation rate was measured by trypan blue exclusion method of cell viability. (**b**). The cells were cotransfected with either miR-346 or control mimics and 200 ng pGL3 reporter construct containing wild-type. The relative firefly luciferase activities normalized with Renilla luciferase were measured 48 h post transfection, and the results are plotted as percentage change over respective controls.(**c**). Tumor cell invasion and angiogenesis were assessed using Matrigel invasion assay and tube formation assay. Representative photomicrographs of in vitro tube formation. We cotransfected miR-346 mimic or mimic control with or without ATV treatment into Raji cells. (**d**) Transcriptional activity of HIF-1α (**$p < 0.005$, *$p < 0.05$). (**e**). Transcriptional activity of VEGF (**$p < 0.005$, *$p < 0.05$). (**f**) Cell

viability: the decreased cell viability induced by miR-346 was restored by HIF-1$\alpha^{\Delta 3'UTR}$ overexpression (**$p < 0.005$, *$p < 0.05$).

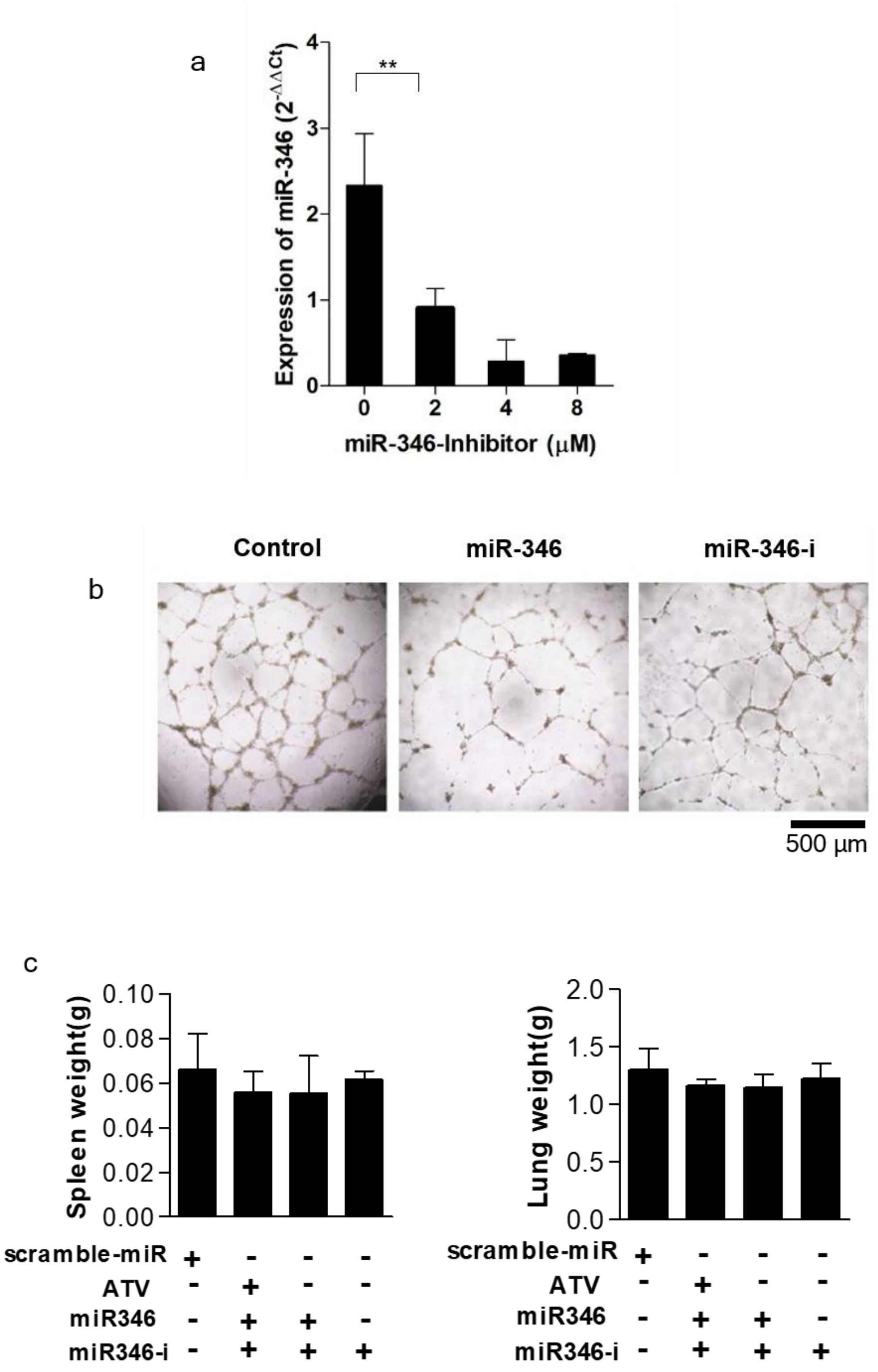

Figure S5. (**a**) The relative expression of miR-346 in Raji cells was analyzed according to the concentration of miR-346 inhibitor by qRT-PCR (**$p < 0.005$). (**b**) Representative photomicrographs of in vitro tube formation after each treatment. (**c**) No weight change in the spleen and lung was detected, clearly indicating absence of acute toxicity.

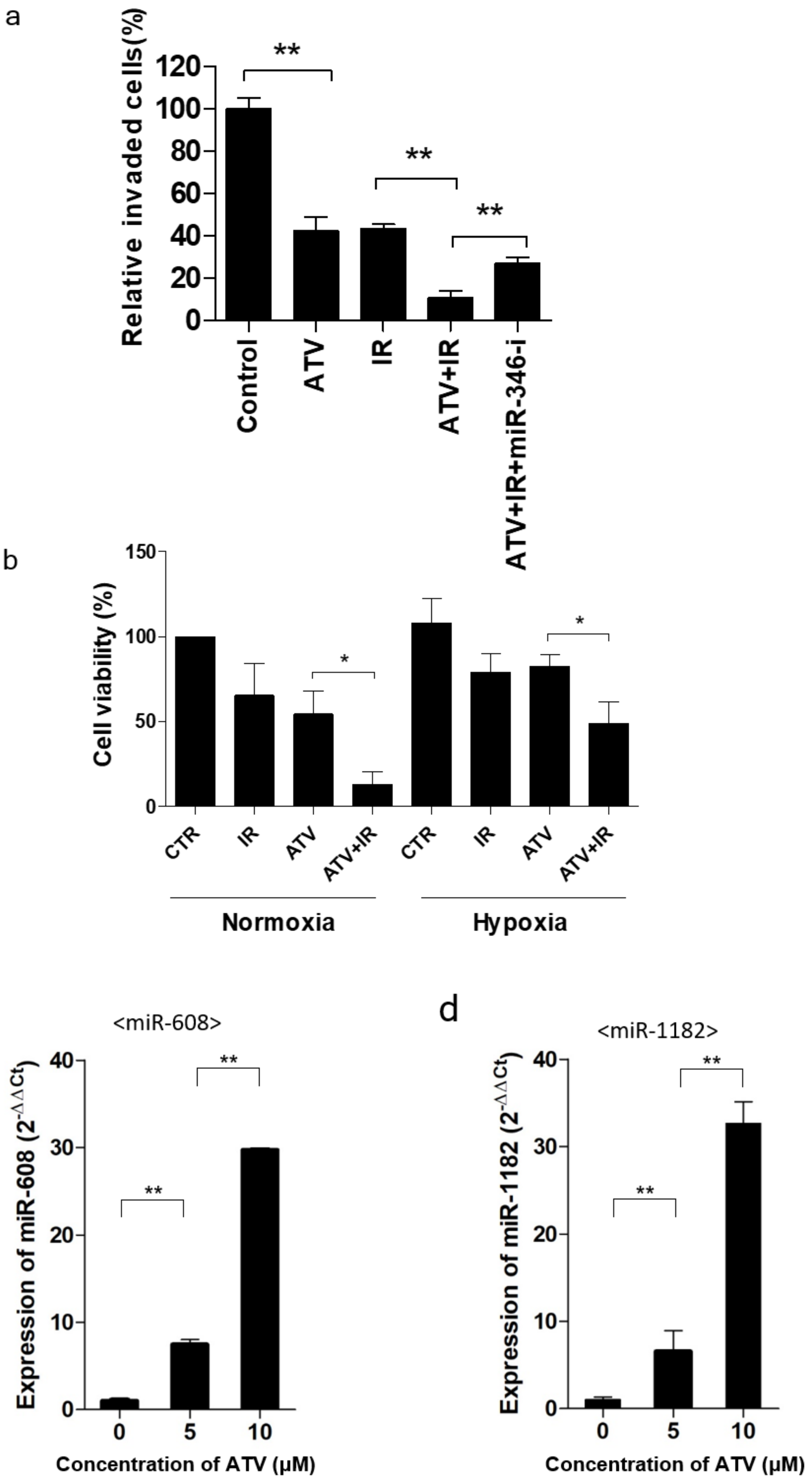
a
Relative invaded cells(%)
120
100
80
60
40
20
0
**
**
**
Control
ATV
IR
ATV+IR
ATV+IR+miR-346-i
b
Cell viability (%)
150
100
50
0
*
*
CTR
IR
ATV
ATV+IR
CTR
IR
ATV
ATV+IR
Normoxia
Hypoxia
c
<miR-608>
Expression of miR-608 ($2^{-\Delta\Delta Ct}$)
40
30
20
10
0
**
**
0
5
10
Concentration of ATV (μM)
d
<miR-1182>
Expression of miR-1182 ($2^{-\Delta\Delta Ct}$)
40
30
20
10
0
**
**
0
5
10
Concentration of ATV (μM)

Figure S6. (**a**) Relative cell tumor invasion (%) by Matrigel invasion assay. (**b**) Cell viability: control and hypoxia-induced Raji cells received irradiation (6 Gy), atorvastatin treatment, or irradiation (6 Gy) + atorvastatin for 48 h. (**c**, **d**) Upregulated expression of miR-1182 and miR-608, which are related to inhibition of tumor growth and induction of cell death.

The whole western blot images of Figure 3, Figure 5a, Figure 6a

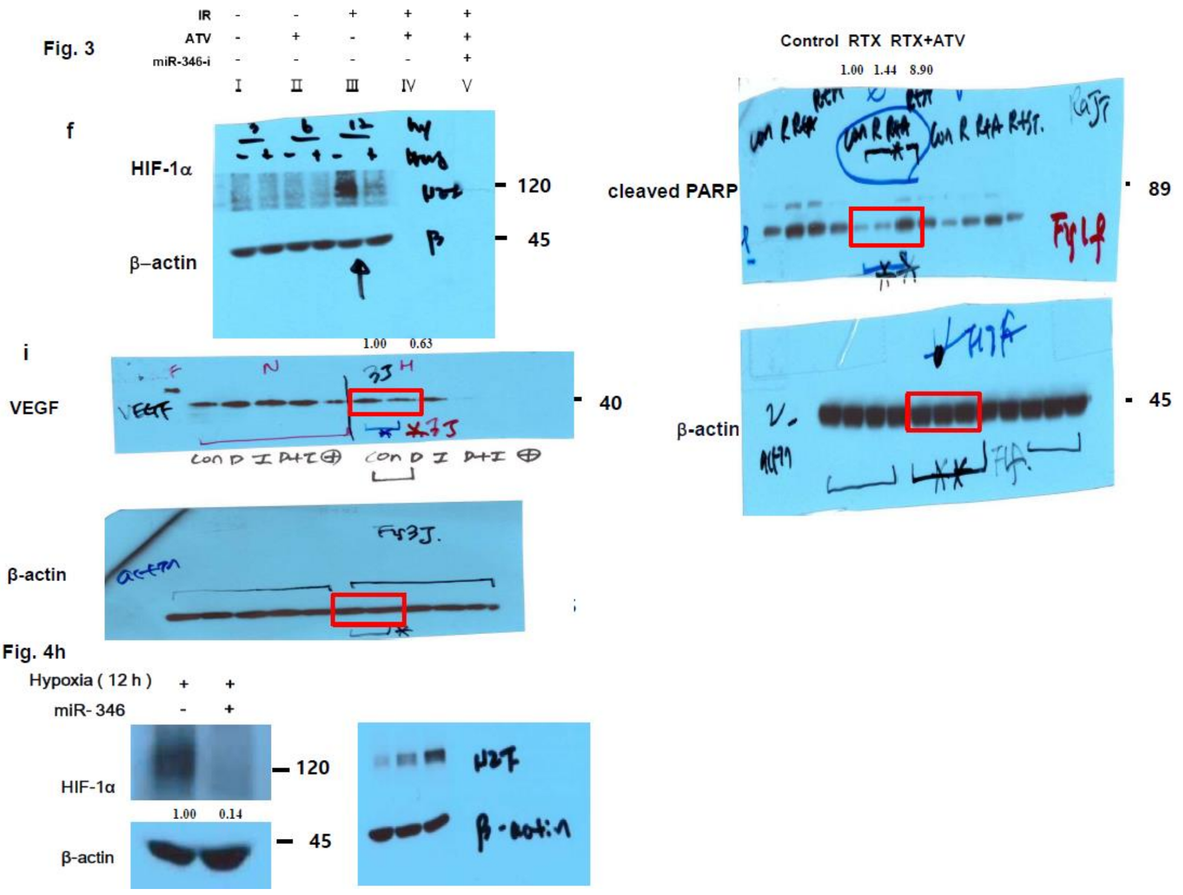

Fig. 5a

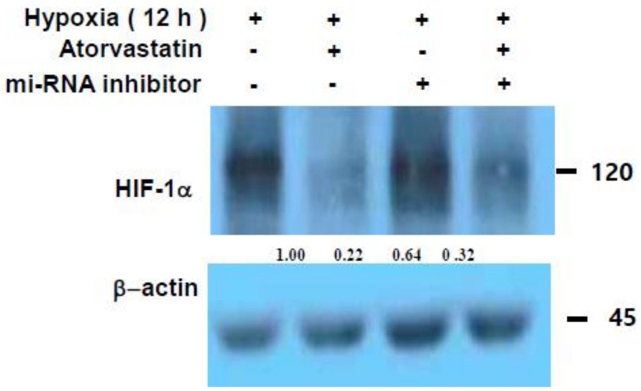

Hypoxia (12 h) + + + +
Atorvastatin - + - +
mi-RNA inhibitor - - + +
HIF-1α
120
1.00 0.22 0.64 0.32
β–actin
45

Fig. 5f

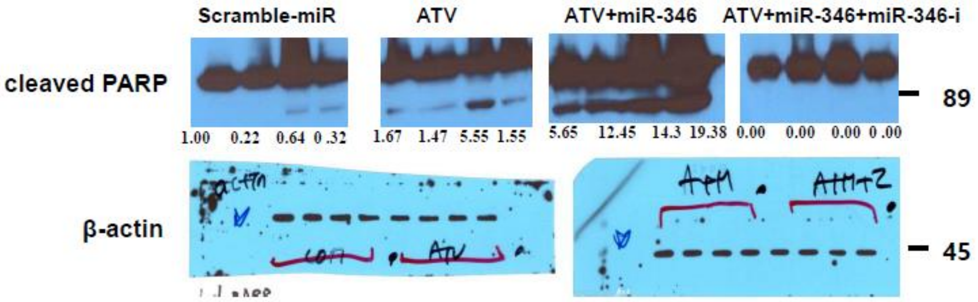

Scramble-miR
ATV
ATV+miR-346
ATV+miR-346+miR-346-i
cleaved PARP
89
1.00 0.22 0.64 0.32 1.67 1.47 5.55 1.55 5.65 12.45 14.3 19.38 0.00 0.00 0.00 0.00
β-actin
45

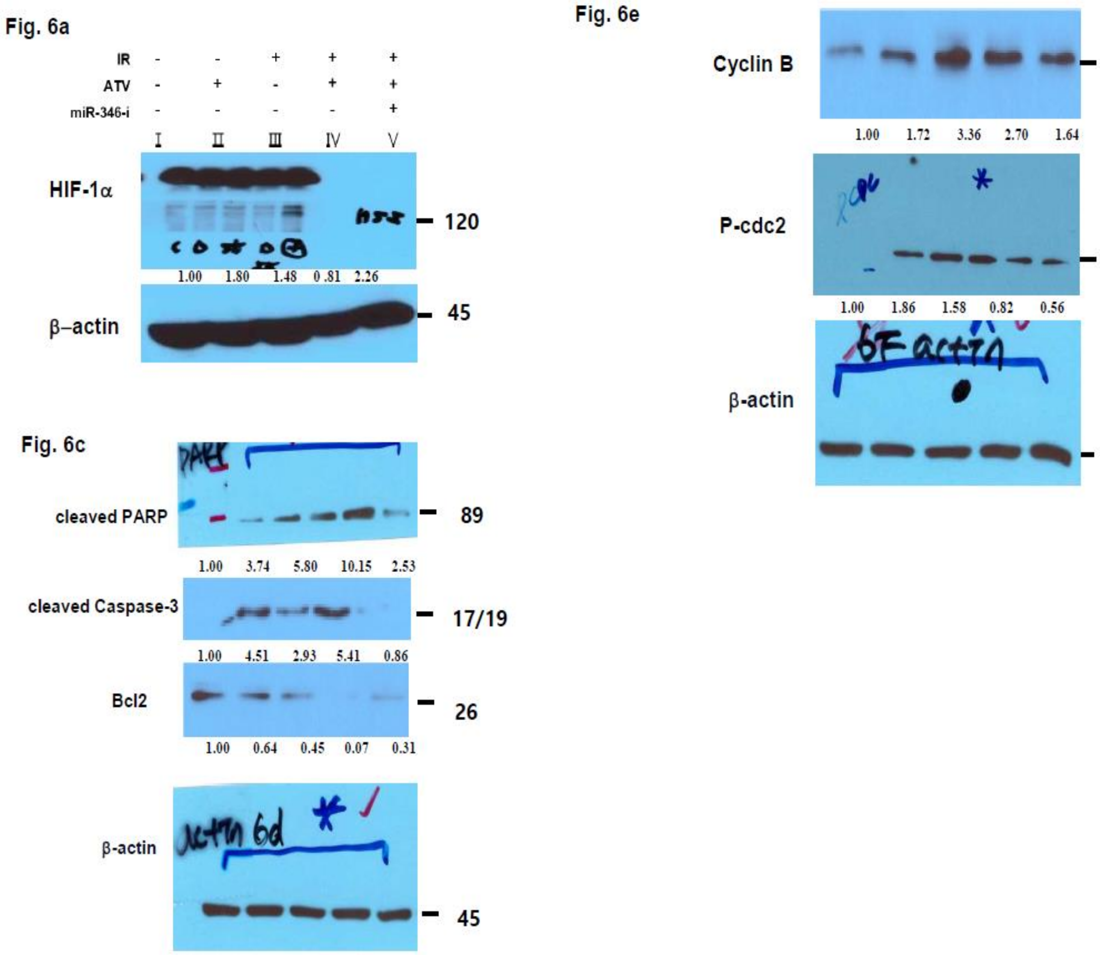

© 2020 by the authors. Submitted for possible open access publication under the terms and conditions of the Creative Commons Attribution (CC BY) license (http://creativecommons.org/licenses/by/4.0/).
